# Supplementary material for: Cortisol response under low intensity exercise during cognitive-behavioral therapy is associated with therapeutic outcome in panic disorder–an exploratory study
Source: PLoS One. 2022 Sep 1;17(9):e0273413. doi: 10.1371/journal.pone.0273413 (PMC9436097; doi:10.1371/journal.pone.0273413)
Supplement: S1 Table — (DOCX) [file pone.0273413.s002.docx]

**Table S1:** Baseline characteristics (sociodemographic and psychopathological variables, BMI) of the N = 20 patients with PD with/ without agoraphobia and the sample of dropped out patients (N = 10).

|  | **Patients with PD with/ without agoraphobia**  **n = 20** | **Drop outs**  **n = 10** | **F/ χ²/ U** | **P** |
| --- | --- | --- | --- | --- |
| Sex, n (%)  females  males | 12 (60)  8 (40) | 7 (70)  3 (30) | .287 | .592(χ²) |
| Age, years median, IQR | 29.8 (22.7-43.3) | 25.0 (22.2-34.6) | 78.000 | .333(U) |
| Familiy status, n (%)  Single/ no partner  Married  cohabitation (not married) | 5 (25)  5 (25)  10 (50) | 5 (45)  1  4 (55) | 2.413 | .491(χ²) |
| Education, n (%)  No graduation  Hauptschule/ Realschule (Secondary School leaving certificate)  High School (Abitur)  University | 0 (0)  9 (45)  7 (35)  4 (20) | 1 (10)^a^  3 (30)  5 (50)  0 (0) | 5.179 | .269(χ²) |
| Body Mass Index (BMI), median (IQR) | 22.2 (20.6-25.1) | 22.9 (21.8-25.7) | 79.000 | .356(U) |
| PAS total score [0-52], median (IQR) | 17.2 (5.9-24.5) | 19.4 (4.0-24.8) | 96.500 | .877 (U) |
| BDI [0-63], median (IQR) | 7.5 (5.3-12.8) | 10.0 (6.0-20.5)^a^ | 71.000 | .370 (U) |

*p ≤ .05, ** p ≤ .01, *** p ≤ .001; ^a^n = 1 missing value; **Abbreviations:** BDI, Beck-Depression-Inventory; BMI, Body Mass Index; IQR, Interquartile Range; PAS, Panic and Agoraphobia Scale
