# Supplementary material for: Cortisol response under low intensity exercise during cognitive-behavioral therapy is associated with therapeutic outcome in panic disorder–an exploratory study
Source: PLoS One. 2022 Sep 1;17(9):e0273413. doi: 10.1371/journal.pone.0273413 (PMC9436097; doi:10.1371/journal.pone.0273413)
Supplement: S1 File — (DOCX) [file pone.0273413.s003.docx]

## A10: Study Protocol

**Study title:**

„Investigating the impact of perceiving odours of differing valence on the neural activation in patients with panic disorder – a study using functional magnetic resonance imaging (fmri)“

**Date of the study protocol:**

05.01.2009

**Corresponding scientist:**

Frau Dr. phil. Dipl.-Psych. Katja Petrowski

Universitätsklinik Carl Gustav Carus an der TU Dresden

Klinik und Poliklinik für Psychotherapie und Psychosomatik

Fetscherstr. 74/ 01307 Dresden

Tel.: (0049) 351/ 458 3634

Fax: (0049) 351/ 458 5713

E-Mail: katja.petrowski@mailbox.tu-dresden.de

**Theoretical background:**

The role of the human olfactory system (e.g. in regulating behavior and emotions) has been widely underestimated (Kohl, Atzmueller, Fink & Grammer, 2001). However, there is numerous evidence showing the impact of olfaction on the central nervous system (z.B. Lorig & Roberts, 1990; Martin, 1996; Jacob & McClintock, 2000; Raudenbush, Koon, Smith, & Zoladz, 2003 Chen, Katdare & Lucas, 2006; Kiecolt-Glaser, Graham, Malarkey, Porter, Lemeshow & Glaser, 2008). Referring to animal models, the behavior-regulating effect of olfactory stimuli has been well proven so far (z.B. Valenta & Rigby, 1968; Carr, Martorano & Krames, 1970; Cocke & Thiessen, 1986; Zalaquett & Thiessen, 1991; Wedekind & Penn, 2000). For instance, the exposition of anxiety-related odours of conspecifics either led to avoidance behavior, increased motor activity or immobility in recipients (Mackay-Sim & Laing, 1981). Following, the perception of odours has an important alarming function and is essential for survival in animals.

Likewise in mankind, the perception of odours of differing valence seems to have an important role in regulating emotions, cognitions and behaviors (z.B. Miltner, Matjak, Braun, Diekmann & Brody, 1994; Moynihan, Karp, Cohen & Ader, 2000; Kikusui, Takigami, Tarkeuchi & Mori, 2001; Pause, Ohrt, Prehn & Ferstl, 2004; Chen, Katdare und Lucas, 2006). In some studies (e.g. Chen & Haviland-Jones, 2000; Ackerl, Atzmueller & Grammer, 2002) it could be shown, that study participants identified odours, segregated during an anxiety-inducing situation (e.g. watching a horror movie), better than odours segregated during neutral or positive situations (e.g. watching a funny movie). Results from cognitive studies (e.g. Pause, Ohrt, Prehn & Ferstl, 2004) and studies using functional magnetic resonance imaging (fmri) (e.g. Sobel, Prabhakaran, Hartley, Desmond, Glover, Sullivan & Gabrieli, 1999) hint towards the automaticity of olfactory processing, as well as the priority towards visual and acoustic processing. Above, by means of functional imaging such as fmri, important neural areas, involved in the neural processing of odours, could be identified, among them bulbus olfactorius, prepyriform cortex, amygdalae, medial dorsal thalamus, anterior gyrus cinguli, insula, hypothalamus, hippocampus, entorhinal cortex, orbitofrontal Cortex (OFC) and lateral posterior orbitofrontal cortex (Eslinger, Damasio & Van Hoesen, 1982; Zald & Pardo, 2000; Rosenzweig, Breedlove & Watson, 2005; Rolls, Grabenhorst, Margot, da Silva & Velazco, 2008). Studies (Andy, Jurko & Hughes, 1975; Zald & Pardo, 1997; Zald & Pardo, 2000; Gottfried, Deichmann, Winston & Dolan, 2002) emphasized the meaning of limbic regions such as the amygdalae, showing by tendency a higher activation under the exposure of unpleasant odours (Zald & Pardo, 1997). Likewise, according to the OFC, different activation patterns could be identified, depending on the valence of the odours (Gottfried et al., 2002). Nevertheless, evidence on this topic remains heterogeneous and ambiguous. Despite of the similarity with other sensory systems, the early involvement of limbic structures by means of the bulbus olfactorius to the amygdalae and the anatomic overlap between the primary olfactory cortex with the anterior part of the amygdalae can be regarded as unique for the olfactory system (Leffingwell, 2002).

The amygdalae and other limbic regions (e.g. hippocampus) play an important role in the etiopathogenesis of panic disorder and other anxiety disorders as well, respectively (Birbaumer, Grodd, Diedrich, Klose, Erb, Lotze, Schneider, Weiss und Flor, 1998; Van den Heuvel, Veltman, Groenewegen, Witter, Merkelbach, Cath, van Balkom, van Oppen & van Dyck, 2005; Etkin & Wager, 2007). However, in panic disorder evidence is also not unambiguous. Moreover, present research could show that patients with panic disorder had a dysfunctional hypothalamus-pituitary-adrenal (HPA) axis (e.g. Ströhle, Holsboer & Rupprecht, 2000). The results on the HPA-axis in panic disorder are heterogeneous as well. (e.g. Bandelow, Wedekind, Sandvoss, Broocks, Hajak, Pauls, Peter & Ruther, 2000; Garcia-Leal, Parenta, Del-Ben, Guimarales, Moreira, Elias & Graeff, 2005). Evidence proving altered corticotropin releasing hormone (CRH) values in patients with panic disorder (e.g. Ströhle et al., 2000) and the fact, that CRH receptors are distributed in the basolateral nucleus of the amygdalae and the hippocampus, lead to the assumption that patients with panic disorder may show an altered perception of odours as well. During a standardized, psychosocial stress test (Trier Social Stress Test/ TSST; Kirschbaum, Pirke & Hellhammer, 1993), including a combination of simulated public speech about one´s personal traits and an arithmetic task, stress hormones are segregated via the HPA-axis and sympathetic-adrenal-medullary (SAM) system. Both stress systems are activated by factors such as social-evaluative threat (Dickerson & Kemeny, 2004), induced uncontrollability and unpredictability of the stress situation and missing feedback. Above, by means of the SAM system, the segregation of sweat is regulated via apocrine glands. The sweat segregated during the above mentioned mental stress situation, is going to be presented to patients with panic disorder with or without agoraphobia. The resulting neural activities during odour presentation is compared to the neural activities of an age- and sex-matched group of healthy controls.

Existing studies on olfactory perception were mainly realized in animals and rather took into account anxiety-related odours as compared to odours from stressful situations (e.g. TSST). Moreover, there is a lack of evidence on the olfactory perception and processing in patients with panic disorder with/ without agoraphobia. Findings from the present study could extend the present knowledge about an altered sensory processing (Ludewig, Geyer, Ramseier, Vollenweider, Rechsteiner & Cattapan-Ludewig, 2005) in patients with panic disorder (e.g. attention bias, memory bias) to the processing of odours. Above, there is evidence, that odours may play an essential role for triggering panic attacks (Hinton, Ba, Peou & Um, 2000; Hinton, Pich & Chhean, 2004; Hinton, Pich, Chhean, Pollack & Barlow, 2004). These insights may be applied in the psychotherapy of panic disorder (e.g. exposure-based therapy using odours, aroma therapy, 1996).

In the present study, the neural activity during the perception of one´s own body odour (sweat odour), produced during a standardized, psychosocial stress situation (TSST), will be investigated and compared with the neural activity under the presentation of a pleasant (peach) or unpleasant (artificial sweat) odour. Additionally, a comparison is realized with the neural activity during the presentation of one´s own body odour (sweat odour), produced during a neutral, physical stress situation (low intensity exercise/ ergometry) (Pause et al., 2004). It is of particular interest, whether patients with panic disorder with/ without agoraphobia differ from healthy controls with respect to their neural activity during the presentation of odours of differing valence.

For the first time, the present study considers olfactory perception in patients with panic disorder with/ without agoraphobia. Above, previous results are retrieved from animal studies or from studies using odours from anxiety-related situations or from studies not applying imaging techniques.

**The following main research questions should be answered by the present study:**

1. Are there differences with respect to the neural activity under the presentation of odours of differing valence (pleasant vs. unpleasant vs. body odour from TSST vs. body odour from low intensity exercise/ ergometry)
2. To what extent the neural activity under the presentation of a pleasant odour differs from the neural activity under the presentation of an unpleasant odour (peach vs. artificial sweat)?
3. To what extent the neural activity under the presentation of one´s own body odour, produced during a standardized, psychosocial stress situation differs from the neural activity under the presentation of one´s own body odour, produced during a neutral physical stress situation (TSST vs. low intensity exercise)?
4. To what extent certain brain areas e.g. amygdalae, hippocampus, orbitofrontal cortex differ with respect to their activity under the presentation of odours of differing valence?
5. Do patients with panic disorder with/ without agoraphobia differ from healthy controls with respect to their neural activity under the presentation of one´s own body odour produced during a standardized, psychosocial stress situation (TSST)?
6. Do patients with panic disorder with/ without agoraphobia differ from healthy controls with respect to their neural activity under the presentation of one´s own body odour produced during low intensity exercise?
7. Do patients with panic disorder with/ without agoraphobia differ from healthy controls with respect to their neural activity under the presentation of a pleasant odour (peach)?
8. Do patients with panic disorder with/ without agoraphobia differ from healthy controls with respect to their neural activity under the presentation of an unpleasant odour (artificial sweat odour)?
9. Do patients with panic disorder with/ without agoraphobia differ from healthy controls with respect to their neural activity in certain brain areas (e.g. amygdalae, hippocampus, orbitofrontal cortex) under the presentation of odours of differing valence (TSST, low intensity exercise, peach, articifical sweat odour) in der Aktivierung der?
10. Which influence do age, gender, cycle week, medication, nicotine consumption, duration of the illness, therapy status have on the neural activity under the presentation of odours of differing valence?

The present study is based on a research project named „Comparing the habituation processes between patients with panic disorder and healthy controls – Examination of the HPA-axis functionality in panic disorder with/ without agoraphobia under hormonal and psychosocial stress“ (number of ethic votum: 46032008). The study participants take part in both studies. Following, a coherent consideration of the stress reactivity and olfactory perception is possible.

**Duration of the present study:**

**Total:** from February 2012 on one year

The standardized, psychosocial stress test has a duration of three hours. The low intensity exercise training has a duration of 30 minutes. The fmri measurement has a duration of 45 minutes. For the present study, a total time expenditure, both for patients and healthy controls, of four hours and 15 minutes may be calculated.

**Study population:** (for the purpose of better illustration see attachment **A9**)

**Recruitment:**

The recruitment of patients with panic disorder with/ without agoraphobia is realized at the Department of Psychotherapy and Psychosomatic Medicine of the University Hospital Carl Gustav Carus Dresden, Technische Universität Dresden. Both, patients from an inpatient, outpatient unit and a day clinic will be enrolled. All study participants who already take part in the ongoing study on habituation processes in patients with panic disorder and healthy controls (see above: EK 46032008) are asked whether they are interested in participating in an additional study on olfactory perception. During the standardized, psychosocial stress test (TSST), patients who were interested in participation in the present study weared a T-shirt free of any odours. Above, an additional appointment for the 30-minute low intensity exercise was arranged. During the training, the study participants also weared a T-shirt, free of any odours. During a further appointment, the fmri measurement took place, with a duration of 45 minutes.

The participants for the healthy control group were recruited by means of newspaper advertisements (e.g. in university magazines, local newspapers), notices in discounters, libraries, intranet and private contacts. All study participants of the healthy control group who already took part in the ongoing study on habituation processes in patients with panic disorder and healthy controls (see above ethic votum EK 46032008) were asked, whether they were interested participating in a further study on odour perception. During the standardized, psychosocial stress test (TSST), the study participants wear a T-shirt free of any odours. Additionally, another appointment is arranged for the 30-minute low intensity exercise. During the latter, the participants also wear a T-shirt free of any odours. During another appointement, the fmri takes place, with a duration of 45 minutes.

The room where the TSST and the low intensity exercise takes place is the same for all study participants. Room temperature and humidity are kept constant. Before the TSST and the low intensity exercise the study participants receive an instruction, to relinquish odour-intensive food (e.g. onion, garlic, cabbage) in the evening before and on the day of the examinations. Above, they are asked to come sober, relinquish smoking, deodorants and other fragances on the testing days. Before testing, the study participants are asked to wash the upper part of their body with an odourless soap.

**Inclusion/ exclusion criteria:**

**Patient group:**

-n=12 patients with a primary diagnosis of panic disorder with/ without agoraphobia (between 18 and 65 years (F 40.01, F 41.0), valid and reliable assessment of the primary diagnosis according to ICD-10 and DSM-IV using DIA-X (computer-assisted DIA-X interview for mental disorders) (Wittchen, Weigel & Pfister, 1996). In case that the standardized assessment of the diagnosis is older than six months, a new standardized assessment is necessary.

-Scondary diagnoses of minor depressive disorders (ICD-10: F32.0) (besides major depressive disorders), secondary diagnosis of problematic alcohol consumption (alcohol abuse) are allowed (ICD-10: F10.1)

In order to preclude morbidity, the following examinations are realized previously, before the present study: structural magnetic resonance tomography (MRT)

**Exclusion criteria:**

Only for patients:

Presence of other secondary anxiety disorders (Social Phobia ICD-10: F40.1), Generalized anxiety disorder (ICD-10: F41.1), obsessive-compulsory disorder (ICD-10: F42.8), posttraumatic stress disorder (PTSD) (ICD-10: F43.1), presence of any psychotic disorder (ICD-10: F20-F29) or bipolar disorder (ICD-10: F31), presence of any major depressive disorder (ICD-10: F32.1, F32.2, F32.3), substance use disorders (ICD-10: F10.2-F16.2, F18.2, F19.2) or eating disorders (ICD-10: F50),

acute infections,

chronic diseases (e.g. metabolic diseases, autoimmune diseases, heart diseases, blood diseases)

current pregnancy

**control groups:**

n=12 healthy controls (matched for age and gender) between 18 and 65 years without acute and chronic medical illnesses

In order to preclude morbidity, the following examinations are realized previously, before the present study: structural magnetic resonance tomography (MRT)

**Exclusion criteria:**

Only for healthy controls:

Current or past diagnosis of panic disorder with/ without agoraphobia (F40.01, F41.0)

Presence of any other anxiety disorder (according to ICD-10: F40.1, F41.1, F42, F43, F42), psychotic (F20-F29) or bipolar disorders (F31), major depression (F32.1, F32.2, F32.3), substance use disorders (F10.2-F16.2, F18.2, F19.2), somatoform disorder (F45.0) or eating disorders (F50),

acute infections,

chronic diseases (e.g. metabolic diseases, autoimmune diseases, heart diseases, blood diseases)

current pregnancy

**Exclusion criteria**

For all:

-diseases of the upper respiratory tract (e.g. viral infectious diseases, allergies with swelling of the nasal mucous membrane

-neurologic diseases (z.B. craniocerbral injury, Parkinson´s disease)

-anosmia (complete loss of the sense of smell) oder hyposmia/ microsmia (partial loss of the sense of smell), Kallmann-syndrome

-medication with impact on the olfactory system (e.g. antihistamine, antidepressives, ACE-inhibitors)

-dissociative disorders

-structural brain damage (seen in MRT), also without a clinical correlate (incidental finding)

-neurostimulation, pacemaker, artificial heart valve, insulin pump or other implants (e.g. cochlear implant)

-metal inside the body (e.g. artificial limbs, vessel clips, metal dust during work)

-tattoos in the region of the head and neck as well as in chest and back area

-fixed retainers

-permanent Make-Up
-artificial limbs and artificial joints
-screwed or nailed fractures

-spirals for contraception (please discuss possible metal with the physician/ gynaecologist)
-grommet (as inserted e.g. in case of inflammation of the middle ear inside the drumhead)
-metal splinter inside tissue

-glasses (can be taken off during the examinations)

-current pregnancy

**Inclusion criteria:**

German as dominant language in case of multilingualism, dexterity

**Calculation of sample size:**

In order to estimate the appropriate sample size, we adhered to a recommendation for fmri studies by Friston, Holmes und Worsley (1999). They recommend at least 12 study participants for the control group and at least 12 study participants for the patient group (patients with panic disorder with/ without agoraphobia).

**Study processes and examinations (For the purpose of better illustration see attachment A1)**

**1. Measuring the neural activity**

Inside the fmri scanner, the study participants are presented odours of differing valence, using an on-off design (see A1).

During the fmri, the study participants smell a pleasant odour (peach), an unpleasant odour (artificial sweat), an odour (own body odour) generated during a psychosocial stress test (anxiety/ strss odour) and an odour (own body odour) generated during a physical stress situation (low intensity exercise/ LIE). The odours will be presented uni-laterally, birhinally by means of a manual olfactometer. We use a design with repeated measure with odour conditions and time as within-subjects factors and group (patients vs. healthy controls) as between-subjects factor (see A1).

Room temperature, humidity and ventilation are held constant throughout the examinations. The study participants receive no information which odour is presented and how often they are presented (Grabenhorst, Rolls, Margot, da Silva & Velazco, 2007). We apply an on-off design, i.e. a certain odour (peach vs. articificial sweat vs. own body odour from psychosocial stress/ anxiety situation vs. own body odour from physical stress/ low intensity exercise) is presented in a determined order (on) or not presented (off). During an on-off sequence, a total of eight scans are measured. A complete on-off sequence (session) consists of twelve sequences. Altogehter, a total of 96 scans are generated per session. In order to preclude order effects during the sessions, the order of odour presentation is balanced between study participants. One sequence lasts 21 seconds. One session lasts four minutes and twelve seconds. The total presentation of all four sequences has a duration of 16 minutes and 48 seconds (see A1). The structural MRI needs additional 20 minutes. The whole time spent in the magnetic resonance tomography is about 40 minutes.

**2. Assessment of psychological variables**

After each odour presentation, the participants are asked which odour was presented, how pleasant the odour was received (valence/ hedonic rating, on a scale ranging from -5 to +5, Grabenhorst et al., 2007) and how intensive it was (intensity rating, on a scale ranging from 0 to 10, Grabenhorst et al., 2007). Additionally, we ask the participants to rate the perceived anxiety when the odour was presented (on a rating scale ranging from 0 to 10, see attachment A1). In previous studies it could be alreay shown that the valence of odour perception is correlated with the neural activity in the amygdalae (e.g. Zald & Pardo, 1997). Additionally, we expect a correlation with the intensity of odour perception, particularly under the presentation of unpleasant odours.

Before and after the fmri examination, the participants are asked according to their State anxiety as assessed using the State- und Trait-Anxiety inventory (STAI Form X-State, Laux, Glanzmann, Schaffner & Spielberger, 1981; Chen, Katdare & Lucas, 2006; A2). The STAI assesses anxiety as trait and state using two scales with 20 items per scale. Anxiety as state is influenced by situational factors, whereas anxiety as trait is a personal, temporally stable characteristic (Trait). Answering the STAI lasts about five minutes.

After finishing the fmri measurement, the participants are informed about the odour presentation. Above further questions concerning the present study participation will be answered. The whole procedure lasts about 45 minutes.

**3. Safe-keeping of data**

The data rised in the present study are kept on computers and steel lockers of the archive located at the department of Psychotherapy and Psychosomatic Medicine. Safe-keeping is in accordance with the existing data protection rules.

**Primary and secondary outcomes:**

-Differences in the neural activity under the presentation of odours of differing valence (primary outcome)

-Differences in the trait and state anxiety between healthy controls and patients with panic disorder with/ without agoraphobia as measured with the STAI (Laux, Glanzmann, Schaffner & Spielberger, 1981) (secondary outcome)

-correlation between valence/ intensity ratings of odours (see attachment A1) and the neural activity (secondary outcome)

**Statistical analyses:**

Basis of the statistical analyses is the comparison between two research groups (patients, healthy controls). The results of the questionnaires (e.g. STAI) have metric data level and are controlled for the presence of normal distribution and the equality of variance. Afterwards, a comparison between groups is realized applying t-tests and analyses of variance for repeated measures, using SPSS version 16.0. The data of the pre-post ratings (see A1) have ordinal data level and are analysed using non-parametric testing (U-test according to Mann and Whitney).

The statistical analyses of the fmri images and the clinical data are realized by way of the interactive programme SPM (Statistical Parametric Imaging) Version 5, after normalization of the data to the anatomical atlas according to MNI (Talairach und Tournoux, 1988) using a pixel-based statistical evaluation. The fmri images are analysed both within the odour conditions and between groups (patients vs. controls) (per odour condition). Above, correlations between frmi data and questionnaire data will be calculated.

**Protection of data privacy:**

The questionnaire data (STAI, pre-post ratings, case report form) and the fmri data are pseudonymized by way of a code number, before entering in a SPSS file that is protected by a password and before statistical analyses. An inspection of data oder dissemination to a third party is excluded. Only project staff has access to personal data. The patients receive a feedback of their study results upon request. In case of the presence of psychological disorders, the patients receive information on treatment options.

**Attachment A1: Test protocol**

| **Odour** | **1-8** | **9-16** | **17-24** | **25-32** | **33-40** | **41-48** | **49-56** | **57-64** | **65-72** | **73-80** | **81-88** | **89-96** |  |
| --- | --- | --- | --- | --- | --- | --- | --- | --- | --- | --- | --- | --- | --- |
| **Peach**  **(positive)** | on | off | on | off | on | off | on | off | on | off | on | off |  |
| **Hydro-gensulfid/ artificial sweat odour**  **(negative)** | on | off | on | off | on | off | on | off | on | off | on | off |  |
| **Body odour/ sweat odour**  **(TSST)** | on | off | on | off | on | off | on | off | on | off | on | off |  |
| **Body Odour/ sweat odour**  **(Low Intensity Exercise)** | on | off | on | off | on | off | on | off | on | off | on | off |  |

| Rating of valence, intensity and perceived anxiety (orally)  STAI-Trait (X2) (Laux et al., 1981)  STAI-G-State (X) (Laux et al., 1981)  on odour is presented  off odour is not presented |
| --- |

**Order of the sessions (P = peach, K = artificial sweat, Kst = sweat odour (Trier Social Stress Test), Ksp = sweat odour (Low Intensity Exercise/ Sports):**

1. **P-K-Kst-Ksp**
2. **K-Kst-Ksp-P**
3. **Kst-Ksp-P-K**
4. **Ksp-P-K-Kst**

**A8: Literatur**

Ackerl, K., Atzmueller, M. & Grammer, K. (2002). The scent of fear. Neuroendocrinology Letters, 23, 79-84.

Andy, O. J., Jurko, M. F. & Hughes, J. R. (1975), The amygdala in relation to olfaction. Confinia Neurologica/ Applied Neurophysiology, 37, 215-222.

Bandelow, B., Wedekind, D., Sandvoss, V., Broocks, A., Hajak, G., Pauls, J., Peter, H. & Ruther, E. (2000). Diurnal variation of cortisol in panic disorder. Psychiatry Research, 95 (3), 245-250.

Bartoshuk, L. M. & Beauchamp, G. K. (1994). Chemical Senses. Annual Review of Psychology, 45, 419-449.

Birbaumer, N., Grodd, W., Diedrich, O., Klose, U., Erb, M., Lotze, M., Schneider, F., Weiss, U. & Flor, H. (1998). fMRI reveals amygdala activation to human faces in social phobics. Neuroreport, 9, 1223-1226.

Brand, G. & Millot, J.L. (2001). Sex differences in human olfaction: between evidence and enigma. The Quarterly Journal of Experimental Psychology B. Comparative and Psychology 54, 259–270.

Carr, W. C., Martorano, R. D. & Krames, L. (1970). Responses of mice to odors associated with stress. Journal of Comparative and Physiological Psychology, 71 (2), 223-228.

Chen, D. & Haviland-Jones, J. (2000). Human olfactory communication of emotion. Perceptual & Motor Skills, 91, 771-781.

Chen, D., Katdare, A. & Lucas, N. (2006). Chemosignals of Fear Enhance Cognitive Performance in Humans. Chemical Senses 31(5), 415-423.

Cocke, R. & Thiessen, D. D. (1986). Chemocommunication among prey and predator species. Animal Learning & Behavior, 14, 90-2.

Dickerson, S.S. & Kemeny, M.E., (2004). Acute stressors and cortisol responses: a theoretical integration and synthesis of laboratory research. Psychological Bulletin, 130 (3), 355-391.

Eslinger, P. J., Damasio, A. R. & Van Hoesen, G. W. (1982). Olfactory dysfunction in man: anatomical and behavioral aspects. Brain and Cognition, 1 (3), 259-285.

Etkin, A. & Wager, T. D. (2007). Functional Neuroimaging of Anxiety: A Meta-Analysis of Emotional Processing in PTSD, Social Anxiety Disorder, and Specific Phobia. American Journal of Psychiatry, 164 (10), 1476-1488.

Friston, K. J., Holmes, A. P. & Worsley, K. J. (1999). Comments And Controversies. How Many Subjects Constitute a Study? NeuroImage, 10, 1-5.

Garcia-Leal, C., Parenta, A., Del-Ben, C., Guimarales, F., Moreira, A., Elias, L. & Graeff, F. (2005). Anxiety and salivary cortisol in symptomatic and nonsymptomatic panic patients and healthy volunteers performing simulated public speaking. Psychiatry Research, 133 (2-3), 239-252.

Gottfried, J. A., Deichmann, R., Winston, J. S. & Dolan, R. J. (2002). Functional Heterogeneity in Human Olfactory Cortex: An Event-Related Functional Magnetic Resonance Imaging Study. The Journal of Neuroscience, 22 (24), 10819-10828.

Hinton, D., Ba, P., Peou, S. & Um, K. (2000). Panic Disorder Among Cambodian Refugees Attending a Psychiatric Clinic. Prevalence and Subtype. General Hospital Psychiatry, 22, 437-444.

Hinton, D., Pich, V. & Chhean, D. (2004). Olfactory-Triggered Panic Attacks Among Khmer Refugees: A Contextual Approach. Transcultural Psychiatry, 41 (2), 155-199.

Hinton, D. E., Pich, V., Chhean, D., Pollack, M. H. & Barlow, D. H. (2004). Olfactory-triggered panic attacks among Cambodian refugees attending a psychiatric clinic. General Hospital Psychiatry, 26, 390-397.

Jacob, S. & McClintock, M. K. (2000). Psychological State and Mood Effects of Steroidal Chemosignals in Women and Men. Hormones and Behavior, 37, 57–78.

Kiecolt-Glaser, J. K., Graham, J. E., Malarkey, W. B., Porter, K., Lemeshow, S. & Glaser, R. (2008). Olfactory influences on mood, autonomic endocrine, and immune function. Psychoneuroendocrinology, 33, 328-339.

Kikusui, T. Takigami, S., Tarkeuchi, Y. & Mori, Y. (2001). Alarm pheromone enhances stress-induced hyperthermia in rats. Physiology & Behavior, 72, 45-50.

Kirschbaum, C., Pirke, K. & Hellhammer, D. (1993). The „Trier Social Stress Test“-A tool for investigating psychobiological stress responses in a laboratory setting. Neuropsychobiology, 28, 76-81.

Kohl, J. V., Atzmueller, M., Fink, B. & Grammer, K. (2001). Human Pheromones: Integrating Neuroendocrinology and Ethology. Neuroendocrinology Letters, 22, 309-321.

Leffingwell, J. C. (2002). Olfaction-Update No. 5. Leffingwell Reports, 2 (1), 1-33.

Lorig, T. S. & Roberts, M. (1990). Odor and cognitive alteration of the contingent negative variation. Chemical Senses, 15, 537-545.

Ludewig, S., Geyer, M. A., Ramseier, M., Vollenweider, F. X., Rechsteiner, E. & Cattapan-Ludewig, K. (2005). Information-processing deficits and cognitive dysfunction in panic disorder. Journal of Psychiatry Neuroscience, 30 (1), 37-43

Mackay-Sim, A. & Laing, D. G. (1981). Rats´ responses to blood and body odors of stressed and non-stressed conspecifics. Physiology & Behavior, 27, 503-510.

Martin, G. N. (1996). Olfactory Remediation: Current Evidence And Possible Applications. Social Science & Medicine, 43 (1), 63-70.

Miltner, W., Matjak, M., Braun, C., Diekmann, H. & Brody, S. (1994). Emotional qualities of odors and their influence on the startle reflex in humans. Psychophysiology, 31, 107-110.

Moynihan, J. A., Karp, J. D., Cohen, N. & Ader, R. (2000). Immun deviation following stress odor exposure: role of endogenous opoids. Journal of Neuroimmunology, 102, 145-153.

Pause, B. M., Ohrt, A., Prehn, A. & Ferstl, R. (2004). Positive Emotional Priming of Facial Perception in Females is Diminshed by Chemosensory Anxiety Signals. Chemical Senses, 29, 797-805.

Raudenbush, B., Koon, J., Smith, J. & Zoladz, P. (2003). Effects of odorant administration on objective and subjective measures of sleep quality, post-sleep mood and alertness, and cognitive performance. North American Journal of Psychology, 5, 181-192.

Rolls, E. T., Grabenhorst, F., Margot, C., da Silva, M. A. A. P. & Velazco, M. I. (2008). Selective Attention to Affective Value Alters How the Brain Processes Olfactory Stimuli. Journal of cognitive Neuroscience, 20 (10), pp. 1815–1826.

Rosenzweig, M. R., Breedlove, S. M. & Watson, N. V (2005). Biological Psychology: An Introduction to Behavioral and Cognitive Neuroscience (4th ed.). (Sunderland, MA: Sinaur Associates, Inc).

Sobel, N., Prabhakaran, V., Hartley, C. A., Desmond, J. E., Glover, G. H., Sullivan, E. V. & Gabrieli, D. E. (1999). Blind smell: brain activation induced by an undetected air-borne chemical. Brain, 122, 209-217.

Ströhle, A., Holsboer & F., Rupprecht, R. (2000). Increased ACTH concentrations associated with cholecystokinin tetrapeptide-induced panic attacks in patients with panic disorder. Neuropsychopharmacology, 22 (3), 251-260.

Umweltbundesamt, 2006. Duftstoffe: Wenn Angenehmes zur Last werden kann. Hintergrundpapier. [Online im Internet: URL: <http://www.umweltdaten.de/publikationen/fpdf-l/3550.pdf> (Stand 14.11.2011)].

Talairach, J. & Tournoux, P. (1988). Co-Planar Stereotaxic Atlas of the Human Brain. Stuttgart: Thieme Verlag.

Valenta, J. G. & Rigby, M. K. (1968). Discrimination of the odor of stressed rats. Science, 161, 599-601.

Van den Heuvel, O. A., Veltman, D. J., Groenewegen, H. J., Witter, M. P., Merkelbach, J., Cath, D. C., van Balkom, A. J. L. M., van Oppen, P. & Van Dyck, R. (2005). Disorder-Specific Neuroanatomical Correlates of Attentional Bias in Obsessive-compulsive Disorder, Panic Disorder, and Hypochondriasis. Archives of General Psychiatry, 62, 922–933.

Wedekind, C. & Penn, D. (2000). MHC genes, body odours, and odour preferences. Nephroloy Dialysis Transplantation, 15, 1269-1271.

Wittchen, H. U., Weigel, A. & Pfister, H. (1996). DIA-X — Diagnostisches Expertensystem. Swets Test Services, Frankfurt.

Yousem, D. M., Maldjian, J. A., Siddiqi, F., Hummel, T., Alsop, D. C., Geckle, R. J., Bilker, W. B. & Doty, R. L. (1999). Gender effects on odor-stimulated functional magnetic resonance imaging. Brain Research, 818, 480-487.

Zalaquett, C. & Thiessen, D. (1991). The effects of odors from stressed mice on conspecific behaviour. Physiology & Behavior, 50, 221, 227.

Zald, D. H. & Pardo, J. V. (1997). Emotion, olfaction, and the human amygdala: Amygdala activation during aversive olfactory stimulation. Proceedings of the National Academy of Sciences USA, 94, 4119-4124.

Zald, D. H. & Pardo. J. V. (2000). Functional neuroimaging of the olfactory system in humans. International Journal of Psychophysiology, 36, 165-181.

**Attachment A9: Study design**

**fMRI**

- **Presentation of odours of different valences using an on-off design (attachment A1)**
- **peach vs. artificial sweat vs. own body odour from TSST vs. own body odour from LIE**

**Low Intensity Exercise (LIE)/ bicycle ergometry**

- **Wearing an odourless T-Shirt**
- **T-Shirt is stored at –80 ° C**

**Initial consultation**

**(information about the study)**

**Diagnostic Interview**

**(short information about the present study)**

**TSST**

**(Trier Social Stress Test)**

- **wearing an odourless T-Shirt**
- **T-Shirt is stored at –80 ° C**

**Interested in a study on stress reactivity and odour perception?**


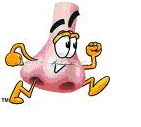


The complete investigation includes four events:

- Psychological stress test during the afternoon at the university hospital Dresden (duration about 2.5 hours), parallely: blood draws, measurement of heart rate and saliva sampling for the determination of the stress hormon cortisol
- examination of the endogenous cortisol secretion (duration about 3 hours), parallely: blood draws
- Low Intensity Exercity (bicycle ergometry of low intensity) (duration about 1 hour), parallely: measurement of heart rate and saliva sampling for the determination of the stress hormon cortisol
- Magnetic Resonance Imaging, parallely: presentation of odours (duration about 1 hour)

**Requirements:** - mentally and somatically healthy participants

- patients with panic disorder

**expense allowance: 150 Euro**

**In case of interest please contact:**

Dr. phil. Katja Petrowski/ Dipl.-Psych. Gloria Wintermann

**Telefon:** **0351/** **458 2079**

**fMRI-study on olfaction: ergometry**

**Date: Code: Polar watch:**

**Please ask patient at arrival to wash him-/ herself with odourless water in the arm pits!**

|  |  | **beginning** | **end** | **notices** |
| --- | --- | --- | --- | --- |
| **Arrival**  (5 min) | **Explain study procedures**  (10 minutes ergometry, before and after saliva samples; two short questionnaires; put on T-Shirt; chest belt and polar watch) |  |  |  |
| **Resting** | **Explain guided breathing**  (5 sec. breathing in, 5 sec. breathing out; 3 min. duration; 6 times (breaths) per minute) |  |  |  |
|  | **Set marker**  Guided breathing at a duration of 3 min. |  |  |  |
| **1. saliva sample**  **-15** | **After guided breathing,** set another marker,  saliva sample 15 min. before Light Intensity Exercise (LIE) (-15)  **~ 3-5 min. before**  **LIE:**  **SAM 1**  **STAI –G 1** |  |  |  |
| **2. saliva sample**  **-1** | **Set marker**  Saliva sample 1 min. before LIE (-1) (15 min. following guided breathing) |  |  |  |
| **LIE/ ergometry**  **10 min** | **Set marker**  **LIE of 10 min. duration** (adjust the height of the bicycle ergometry; intensity level 10 Watt; heart rate at least 110 bpm, 120 bpm at maximum) |  |  |  |
| **3. saliva sample**  **+1** | **Set marker**  Saliva sample 1 min. after LIE, two short questionnaires  (+1)  **SAM 2**  **STAI –G 2** |  |  |  |
| **4. saliva sample**  **+10** | **Set marker**  **Saliva sample 10** min. after LIE  (+10) |  |  |  |
| **5. saliva sample**  **+20** | **Set marker**  20 min. after LIE  (+20) |  |  |  |
| **6. saliva sample**  **+30** | **Set marker**  30 min. after LIE  (+30) |  |  |  |
| **discharge** | Put off polar watch/ chest belt; put off T-shirt |  |  |  |
|  | Discharge of patient |  |  |  |
|  | Contact patient via telephone for a fMRI appointment (suggest an appointment; give hint that respiratory illnesses preclude study participation) |  |  |  |

**Displayed is the procedure of ergometry using LIE (Low Intensity Exercise)**

SAM = Self Assessment Manikin; STAI = State Trait Anxiety Inventory

**- hint:** leave saliva sample at least 20 seconds inside the mouth, chew on the saliva sample until it is sufficiently insalivated
