# Supplementary material for: Cortisol response under low intensity exercise during cognitive-behavioral therapy is associated with therapeutic outcome in panic disorder–an exploratory study
Source: PLoS One. 2022 Sep 1;17(9):e0273413. doi: 10.1371/journal.pone.0273413 (PMC9436097; doi:10.1371/journal.pone.0273413)
Supplement: S2 File — (DOCX) [file pone.0273413.s004.docx]

## A10: Prüfplan

**Studientitel:**

„Untersuchung des Einflusses der olfaktorischen Wahrnehmung unterschiedlich valenter Geruchsreize auf die neuronale Aktivierung bei Patienten mit Panikstörung - eine fMRT-Studie“

**Datum des Prüfplans:**

05.01.2009

**Ansprechpartner:**

Frau Dr. phil. Dipl.-Psych. Katja Petrowski

Universitätsklinik Carl Gustav Carus an der TU Dresden

Klinik und Poliklinik für Psychotherapie und Psychosomatik

Fetscherstr. 74/ 01307 Dresden

Tel.: 0351/ 458 3634

Fax: 0351/ 458 5713

E-Mail: katja.petrowski@mailbox.tu-dresden.de

**Theoretische Grundlagen:**

Die Rolle des olfaktorischen Systems beim Menschen wurde bisher weitgehend unterschätzt (Kohl, Atzmueller, Fink & Grammer, 2001), gleichwohl es zahlreiche Belege für den Einfluss von Geruchsreizen auf das menschliche Zentralnervensystem gibt (z.B. Lorig & Roberts, 1990; Martin, 1996; Jacob & McClintock, 2000; Raudenbush, Koon, Smith, & Zoladz, 2003 Chen, Katdare & Lucas, 2006; Kiecolt-Glaser, Graham, Malarkey, Porter, Lemeshow & Glaser, 2008). Am Tiermodell wurde die verhaltensregulierende Wirkung olfaktorischer Reize bereits gut belegt (z.B. Valenta & Rigby, 1968; Carr, Martorano & Krames, 1970; Cocke & Thiessen, 1986; Zalaquett & Thiessen, 1991; Wedekind & Penn, 2000). Die Exposition mit dem angstbesetzten Geruch von Artgenossen führte dabei bei den Empfängern entweder zu Vermeidungsverhalten, erhöhter motorischer Aktivität oder Immobilität (Mackay-Sim & Laing, 1981). Die Wahrnehmung von Gerüchen hat demnach eine wichtige Signalfunktion und ist für das Überleben im Tierreich von essentieller Bedeutung.

Auch beim Menschen scheint die Wahrnehmung von unterschiedlich valenten Gerüchen eine wichtige emotions-, kognitions- und verhaltensregulierende Rolle zu spielen (z.B. Miltner, Matjak, Braun, Diekmann & Brody, 1994; Moynihan, Karp, Cohen & Ader, 2000; Kikusui, Takigami, Tarkeuchi & Mori, 2001; Pause, Ohrt, Prehn & Ferstl, 2004; Chen, Katdare und Lucas, 2006). In Studien (Chen & Haviland-Jones, 2000; Ackerl, Atzmueller & Grammer, 2002) konnte dabei gezeigt werden, dass Gerüche, die während einer angstbesetzten Situation (z.B. einen Horrorfilm anschauen) abgesondert werden, von den Versuchsteilnehmern besser identifiziert werden können als Gerüche aus neutralen oder positiven Situationen (z.B. einen lustigen Film anschauen). Ergebnisse aus kognitiven Studien (z.B. Pause, Ohrt, Prehn & Ferstl, 2004) und Studien mit bildgebenden Verfahren (z.B. Sobel, Prabhakaran, Hartley, Desmond, Glover, Sullivan & Gabrieli, 1999) weisen dabei auf die Automatizität bei der Verarbeitung olfaktorischer Reize sowie die Priorität gegenüber visuellen oder akustischen Materials hin. Weiterhin konnten mit bildgebenden Verfahren wie der funktionellen Magnetresonanztomografie (fMRT) wesentliche Areale, die an der Verarbeitung olfaktorischer Reize beteiligt sind, identifiziert werden, darunter Bulbus Olfactorius, Präpyriform Cortex, Amygdala, Medialer dorsaler Thalamus, Anteriorer Gyrus Cinguli, Insula, Hypothalamus, Hippocampus, entorhinaler Cortex, Orbitofrontaler Cortex (OFC) und Lateraler posteriorer orbitofrontaler Cortex (Eslinger, Damasio & Van Hoesen, 1982; Zald & Pardo, 2000; Rosenzweig, Breedlove & Watson, 2005; Rolls, Grabenhorst, Margot, da Silva & Velazco, 2008). Studien (Andy, Jurko & Hughes, 1975; Zald & Pardo, 1997; Zald & Pardo, 2000; Gottfried, Deichmann, Winston & Dolan, 2002) haben dabei die Bedeutung limbischer Regionen wie z.B. der Amygdala hervorgehoben, die tendenziell stärker bei der Darbietung unangenehmer Gerüche aktiviert ist (Zald & Pardo, 1997). Auch für den OFC konnten je nach Valenz der Geruchsreize unterschiedliche Aktivitätsmuster festgestellt werden (Gottfried et al., 2002). Die Befundlage hierzu ist allerdings inkonsistent und uneindeutig. Trotz Ähnlichkeit mit anderen sensorischen Verarbeitungssystemen ist die frühe Involviertheit limbischer Strukturen über den Bulbus Olfactorius zur Amygdala sowie die anatomische Überlappung zwischen dem Primären Olfaktorischen Cortex mit dem anterioren Teil der Amygdala dem olfaktorischen System einzigartig (Leffingwell, 2002).

Die Amygdala und andere limbische Regionen (z.B. Hippocampus) spielen auch bei Patienten mit einer Panikstörung bzw. auch bei anderen Angststörungen eine wesentliche Rolle (Birbaumer, Grodd, Diedrich, Klose, Erb, Lotze, Schneider, Weiss und Flor, 1998; Van den Heuvel, Veltman, Groenewegen, Witter, Merkelbach, Cath, van Balkom, van Oppen & van Dyck, 2005; Etkin & Wager, 2007), gleichwohl die Befundlage auch hier nicht ganz eindeutig ist. Weiterhin ist aus bisherigen Studien bekannt, dass Personen mit einer Panikstörung Dysfunktionen der Hypothalamus-Hypophysen-Nebennierenrinden-Achse (HHNA) aufweisen (z.B. Ströhle, Holsboer & Rupprecht, 2000). Die Ergebnisse hierzu sind allerdings ebenso heterogen (z.B. Bandelow, Wedekind, Sandvoss, Broocks, Hajak, Pauls, Peter & Ruther, 2000; Garcia-Leal, Parenta, Del-Ben, Guimarales, Moreira, Elias & Graeff, 2005). Hinweise auf veränderte CRH-Werte bei Patienten mit einer Panikstörung (z. B. Ströhle et al., 2000) und die Tatsache, dass Corticotropin-Releasing-Hormon (CRH)-Rezeptoren sich u. a. auch im basolateralen Nucleus der Amygdala und im Hippocampus befinden, lässt vermuten, dass Patienten mit einer Panikstörung auch Veränderungen der Geruchsreizwahrnehmung aufweisen könnten. Während einer standardisierten, psychosozialen Stresssituation (Trierer Sozial-Stress Test; Kirschbaum, Pirke & Hellhammer, 1993), die eine Kombination aus einer simulierten öffentlichen Rede zu persönlichen Eigenschaften und einer arithmetischen Aufgabe beinhaltet, kommt es zur Ausschüttung von Stresshormonen über die HHN-Achse und das sympathico-adreno-medulläre System (SAM). Beide Stresssysteme werden über Faktoren wie sozial-evaluative Bedrohung (Dickerson & Kemeny, 2004), induzierte Unkontrollierbarkeit und Unvorhersagbarkeit der Situation durch fehlende Rückmeldung aktiviert. Über das SAM wird auch die Schweißabsonderung über apokrine Drüsen reguliert. Der in dieser mentalen Belastungssituation produzierte Körperschweiß soll in der vorliegenden Studie Patienten mit einer Panikstörung mit bzw. ohne Agoraphobie dargeboten werden und die resultierende Hirnaktivation während der Geruchsreizdarbietung mit der Hirnaktivation der nach Alter und Geschlecht parallelisierten Gruppe gesunder Personen verglichen werden.

Bisherige Studien zur olfaktorischen Wahrnehmung wurden v. a. am Tiermodell vorgenommen und berücksichtigten eher angstbesetzte Gerüche im Vergleich zu Gerüchen aus stressreichen Situationen (z.B. TSST). Zudem fehlen bisher Befunde zur Geruchsreizwahrnehmung bei Patienten mit Panikstörung mit bzw. ohne Agoraphobie. Erkenntnisse aus der vorliegenden Studie würden Defizite bzw. Veränderungen in der Informationsverarbeitung (Ludewig, Geyer, Ramseier, Vollenweider, Rechsteiner & Cattapan-Ludewig, 2005) bei Patienten mit einer Panikstörung (z.B. Aufmerksamkeits-Bias, Gedächtnis-Bias) auf die Verarbeitung von Geruchsreizen ausweiten. Zudem gibt es Befunde, die zeigen konnten, dass Gerüche bei der Auslösung von Panikattacken eine wesentliche Rolle spielen können (Hinton, Ba, Peou & Um, 2000; Hinton, Pich & Chhean, 2004; Hinton, Pich, Chhean, Pollack & Barlow, 2004). Diese Erkenntnisse könnten auch für die therapeutische Behandlung nutzbar gemacht werden (z.B. Konfrontationsbehandlung mit Gerüchen; Aromatherapie, Martin, 1996).

In der vorliegenden Studie soll daher die Hirnaktivation bei der Wahrnehmung von eigenem Körperschweißgeruch, der während einer standardisierten, psychosozialen Stresssituation (TSST) produziert wird, untersucht und mit der Aktivation bei der Darbietung eines positiven (Pfirsich) bzw. negativen (Künstlicher Schweiß) Geruchs sowie der Darbietung von eigenem Körperschweißgeruch aus einer neutralen, physischen Belastungssituation (Fahrradergometrie, Pause et al., 2004) verglichen werden. Dabei interessiert v. a., ob sich Patienten mit einer Panikstörung mit bzw. ohne Agoraphobie von gesunden Kontrollpersonen hinsichtlich der Hirnaktivation während der Darbietung unterschiedlich valenter Geruchsbedingungen unterscheiden.

Die vorliegende Studie betrachtet erstmals die Geruchswahrnehmung von Patienten mit Panikstörung mit bzw. ohne Agoraphobie. Zudem liegen bisherige Befunde nur aus Tierstudien vor oder nutzten Gerüche aus angstbesetzten Situationen ohne bildgebende Verfahren.

**Folgende Hauptfragestellungen sollen durch die vorliegende Studie geklärt werden:**

1. Gibt es Unterschiede in der neuronalen Aktivierung bei der Darbietung unterschiedlich valenter Geruchsreize (positiv vs. negativ vs. Körperschweißgeruch TSST vs. Körperschweißgeruch Fahrradergometrie)?
2. Inwiefern unterscheidet sich die neuronale Aktivierung bei der Darbietung eines positiven Geruchs von der neuronalen Aktivierung bei der Darbietung eines negativen Geruchs (Pfirsich vs. Künstlicher Schweiß)?
3. Inwiefern unterscheidet sich die neuronale Aktivierung bei der Darbietung von Körperschweißgeruch aus einer standardisierten, psychosozialen Belastungssituation von der neuronalen Aktivierung bei der Darbietung von Körperschweißgeruch aus einer neutralen physischen Belastungssituation (TSST vs. Fahrradergometrie)?
4. Inwiefern unterscheiden sich Amygdala, Hippocampus und Orbitofrontaler Cortex hinsichtlich der Aktivierung bei der Darbietung unterschiedlich valenter Gerüche?
5. Unterscheiden sich Patienten mit/ ohne Agoraphobie von gesunden Kontrollpersonen in der neuronalen Aktivierung bei der Darbietung von eigenem Körperschweißgeruch aus einer standardisierten, psychosozialen Stresssituation (TSST)?
6. Unterscheiden sich Patienten mit/ ohne Agoraphobie von gesunden Kontrollpersonen in der neuronalen Aktivierung bei der Darbietung von Körperschweißgeruch aus einer neutralen, physischen Belastungssituation?
7. Unterscheiden sich Patienten mit/ ohne Agoraphobie von gesunden Kontrollpersonen in der neuronalen Aktivierung bei der Darbietung von positiv valentem Geruch (Pfirsich)?
8. Unterscheiden sich Patienten mit/ ohne Agoraphobie von gesunden Kontrollpersonen in der neuronalen Aktivierung bei der Darbietung von negativ valentem Geruch (Künstlicher Schweiß)?
9. Inwiefern unterscheiden sich Patienten mit/ ohne Agoraphobie von gesunden Kontrollpersonen bei der Darbietung unterschiedlich valenter Gerüche (TSST, Fahrradergometrie, Pfirsich, Künstlicher Schweiß) in der Aktivierung der Amygdala, Hippocampus und des Orbitofrontalen Cortex?
10. Welchen Einfluss haben Alter, Geschlecht, Zyklusstatus, Medikation, Nikotinkonsum, Erkrankungsdauer und Therapiestadium auf die neuronale Aktivierung bei der Darbietung unterschiedlich valenter Gerüche?

Diese Studie steht in Kooperation mit dem Forschungsprojekt „Vergleich der Habituationsprozesse zwischen Panikern sowie Gesunden - Untersuchung der HPA-Achsen- Funktionalität bei Panikstörung mit und ohne Agoraphobie bei hormonell und psychosozial induziertem Stress“ (Ethikantragsnummer: 46032008). Die Versuchsteilnehmer nehmen an beiden Studien teil. Eine zusammenhängende Betrachtung der Stressreaktivität und Geruchswahrnehmung ist somit möglich.

**Studiendauer:**

**Gesamt:** Februar 2012 bis Februar 2013

Die Durchführung des standardisierten, psychosozialen Stresstest dauert etwa 3 Stunden. Das Fahrradergometer-Training dauert etwa 30 Minuten. Die fMRT-Messung dauert etwa 45 Minuten. Der gesamte zeitliche Aufwand sowohl für die Patienten als auch für die gesunden Kontrollpersonen würde sich für die vorliegende Studie auf ca. 4 Stunden 15 Minuten belaufen.

**Studienpopulation:** (Zur Veranschaulichung siehe Anlage **A9**)

**Rekrutierung:**

Die Rekrutierung der Patienten mit Panikstörung mit bzw. ohne Agoraphobie erfolgt über die Klinik und Poliklinik für Psychotherapie und Psychosomatik des Universitätsklinikums Dresden. Es werden stationäre, teilstationäre und ambulante Patienten rekrutiert. Alle Versuchsteilnehmer der bereits laufenden Studie zu Habituationsprozessen bei Patienten mit Panikstörung und Gesunden (EK 46032008) werden befragt, ob sie an einer zusätzlichen Studie zur Geruchsreizwahrnehmung teilnehmen möchten. Bei der Durchführung des standardisierten, psychosozialen Belastungstest (TSST) tragen die Versuchsteilnehmer, die an einer Teilnahme interessiert sind, ein geruchsneutrales T-Shirt. Zudem wird ein zusätzlicher Termin vereinbart für ein 30-minütiges Fahrradergometer-Training. Während des Trainings tragen die Versuchsteilnehmer ebenfalls ein geruchsneutrales T-Shirt. An einem weiteren Termin erfolgt die etwa 45-minütige fMRT-Messung.

Die Untersuchungsteilnehmer der Kontrollgruppe werden per Zeitungsannonce (Studentenzeitung, Lokalzeitung), Aushänge (Kaufland, Bibliotheken), Intranet sowie im privaten Umfeld rekrutiert. Alle Versuchsteilnehmer der Kontrollgruppe der bereits laufenden Studie zu Habituationsprozessen bei Patienten mit Panikstörung und Gesunden (EK 46032008) werden befragt, ob sie an einer zusätzlichen Studie zur Geruchsreizwahrnehmung teilnehmen möchten. Bei der Durchführung des standardisierten, psychosozialen Belastungstest (TSST) tragen die Versuchsteilnehmer, die an einer Teilnahme interessiert sind, ein geruchsneutrales T-Shirt. Zudem wird ein zusätzlicher Termin vereinbart für ein 30-minütiges Fahrradergometer-Training. Während des Trainings tragen die Versuchsteilnehmer ebenfalls ein geruchsneutrales T-Shirt. An einem weiteren Termin erfolgt die etwa 45-minütige fMRT-Messung.

Der Raum zur Durchführung des TSST sowie für das Fahrradergometer-Training wird für alle Versuchsteilnehmer konstant gehalten. Raumtemperatur und Luftfeuchtigkeit werden jeweils kontrolliert. Die Versuchsteilnehmer werden vor dem TSST und dem Fahrradergometer-Training instruiert, am Abend vorher bzw. am Untersuchungstag keine geruchsintensiven Nahrungsmittel zu sich zu nehmen (z.B. Zwiebel, Knoblauch, Kohl), nüchtern zur Testung zu kommen, vorher nicht zu rauchen und auch keine Deodorants oder sonstige Duftstoffe zu verwenden. Vor der Versuchsdurchführung sollen die Versuchsteilnehmer ihren Oberkörper mit einer geruchsneutralen Seife waschen.

**Ein- und Ausschlusskriterien:**

**Patientengruppe:**

n=12 primäre Panikstörung **mit/ ohne Agoraphobie** zwischen 18 und 65 Jahren (F 40.01, F 41.0, valide, reliable und standardisierte Erfassung der Hauptdiagnose nach ICD-10 und DSM-IV mit dem DIA-X (Wittchen, Weigel & Pfister, 1996)). Falls die standardisierte Diagnostik länger als 6 Monate zurückliegt bis die Untersuchung durchgeführt wird, muss erneut eine standardisierte Diagnostik erfolgen

sekundär dürfen leichte depressive Störungen (außer Major Depression) vorliegen (F32.0), sekundär darf Alkoholmissbrauch vorliegen (F10.1)

Zum Ausschluss krankhafter Befunde im Vorfeld der fMRT-Studie werden folgende studienbedingte Untersuchungen durchgeführt: strukturelles MRT

**Ausschlusskriterien:**

Nur für Patienten:

Vorliegen anderer sekundärer Angststörungen (Soziale Phobie (F40.1), Generalisierte Angststörung (F41.1), Zwangsstörung (F42.8), PTSD (F43.1)), Vorliegen einer psychotischen (F20-F29) oder bipolaren Erkrankung (F31), Vorliegen einer Major Depression (F32.1, F32.2, F32.3), Substanzabhängigkeit (F10.2-F16.2, F18.2, F19.2) oder Essstörung (F50),

keine akuten Infektionen,

keine chronischen Erkrankungen (Stoffwechselerkrankungen, Autoimmunerkrankungen, Herzerkrankungen, Bluterkrankungen)

**Kontrollgruppe:**

n=12 gesunde Kontrollpersonen (nach Alter und Geschlecht gematcht) zwischen 18 und 65 Jahren ohne akute und chronische Erkrankungen

Zum Ausschluss krankhafter Befunde im Vorfeld der fMRT-Studie werden folgende studienbedingte Untersuchungen durchgeführt: strukturelles MRT

**Ausschlusskriterien:**

Nur für Kontrollpersonen:

derzeitige oder vorangegangene Panikstörung mit/ ohne Agoraphobie (F40.01, F41.0)

Vorliegen einer anderen Angststörung (F40.1, F41.1, F42, F43, F42), psychotischen (F20-F29) oder bipolaren Erkrankung (F31), Major Depression (F32.1, F32.2, F32.3), Substanzabhängigkeit (F10.2-F16.2, F18.2, F19.2), Somatisierungsstörung (F45.0) oder Essstörung (F50),

keine akuten Infektionen,

keine chronischen Erkrankungen (Stoffwechselerkrankungen, Autoimmunerkrankungen, Herzerkrankungen, Bluterkrankungen)

**Ausschlusskriterien:**

Für alle:

-Erkrankungen der oberen Atemwege (z.B. Viruserkrankung, Allergie mit Schwellung der Nasenschleimhäute, Sinusitis, Rhinitis) oder andere Atemwegserkrankungen

-Neurologische Erkrankungen (z.B. Schädel-Hirn-Trauma, Morbus Parkinson)

-Anosmie (vollständiger Verlust des Geruchssinnes) oder Hyposmie (teilweiser Verlust des Geruchssinnes), Kallmann-Syndrom

-Medikamente mit Auswirkungen auf das olfaktorische System (z.B. Antihistamine, Antidepressiva, ACE-Hemmer)

-Dissoziative Störung

-Struktureller Hirnschaden im MRT, auch ohne klinisches Korrelat (Zufallsbefund)

-Neurostimulatoren, Herzschrittmacher, künstliche Herzklappe, Insulinpumpe oder andere Implantate (z.B. Chochleaimplantate)

-Metall im Körper (Prothesen, Gefäßclips, Metallstaub aus beruflicher Tätigkeit)

-Tätowierungen im Kopf- und Nackenbereich, sowie dem oberen Brust- und Rückenbereich

-Feste Zahnspangen

-permanent Make-Up
-Prothesen und künstliche Gelenke
-geschraubte oder genagelte Knochenbrüche

-Spiralen zur Empfängnisverhütung (bitte mögliche Metallanteile mit dem Arzt abklären)
-Paukenröhrchen (werden z.B. bei Mittelohrentzündung ins Trommelfell eingesetzt)
-Metallsplitter im Gewebe

-Brille

**Einschlusskriterien:**

Deutsch ist die dominante Sprache, falls Mehrsprachigkeit vorliegt

Rechtshändigkeit

**Fallzahlbestimmung:**

Zur Abschätzung der Stichprobengröße wurde einer Empfehlung von Friston, Holmes und Worsley (1999) für funktionell-bildgebende Studien gefolgt, die mindestens 12 Teilnehmer für die Kontrollgruppe und mindestens 12 Teilnehmer für die Patientengruppe (Panikpatienten mit/ ohne Agoraphobie) empfehlen.

**Studienablauf und Untersuchungsmethoden: (Zur Veranschaulichung: A1)**

**1. Messung neuronalen Aktivierung**

Im fMRT-Scanner werden den Versuchsteilnehmern unterschiedlich valente Geruchsreize im on-off Design dargeboten (siehe A1).

Den Versuchsteilnehmern werden bei der fMRT-Messung ein Geruch mit positiver Valenz (Pfirsich), ein Geruch mit negativer Valenz (Künstlicher Schweiß), ein Geruch aus einer psychosozialen Stresssituation (eigener Körperschweiß) sowie eigener Körperschweiß aus physischer Aktivität (Ergometer-Training) dargeboten. Die Gerüche werden nicht-lateralisiert, birhinal über ein handbetriebenes Olfaktometer präsentiert. Es ergibt sich ein Messwiederholungsdesign mit den Geruchsbedingungen und der Zeit als Innersubjektfaktoren sowie der Gruppe (Patienten vs. Gesunde Personen) als Zwischensubjektfaktor (siehe A1).

Raumtemperatur, Luftfeuchtigkeit und Ventilation werden konstant gehalten. Die Versuchspersonen bekommen vorher keine Informationen, welche Gerüche wann und wie oft dargeboten werden (Grabenhorst, Rolls, Margot, da Silva & Velazco, 2007). Es wird ein on-off Design realisiert, d.h. ein bestimmter Geruch (Pfirsich vs. Künstlicher Schweiß vs. Körperschweiß-Stress vs. Körperschweiß-Sport) wird in festgelegter Reihenfolge dargeboten (on) bzw. nicht dargeboten (off). In einer on- bzw. off-Sequenz werden insgesamt acht Bilder erhoben. Eine vollständige on-off-Reihe (Session) besteht aus 12 Sequenzen. Insgesamt werden demnach pro Session 96 Bilder generiert. Um Reihenfolgeeffekte der Sequenzreihen (Sessions) zu vermeiden, wird die Reihenfolge zwischen den Versuchsteilnehmern ausbalanciert. Eine Sequenz dauert etwa 21 Sekunden. Eine Session dauert etwa 4 Minuten 12 Sekunden. Insgesamt dauert die Darbietung aller vier Sequenzreihen etwa 16 Minuten und 48 Sekunden (siehe A1). Die strukturelle Messung würde noch mal 20 Minuten in Anspruch nehmen, sodass sich die gesamte im Scanner verbrachte Zeit auf etwa 40 Minuten beläuft.

**2. Erfassung psychologischer Variablen**

Nach jeder Geruchsdarbietung wird mündlich über eine Fernsprechanlage erfragt, welcher Geruch dargeboten wurde, als wie angenehm (Valenz/ Hedonik, Skala von -5 bis +5, Grabenhorst et al., 2007) bzw. wie intensiv (Intensität, Skala von 0 bis 10, Grabenhorst et al., 2007) dieser empfunden wurde und wie stark die wahrgenommene Angst war (Skala von 0 bis 10, siehe A1). Hier zeigte sich bereits in vorangegangenen Studien, dass die wahrgenommene Valenz mit der Höhe der Amygdala-Aktivierung korreliert (z.B. Zald & Pardo, 1997), wobei auch eine Korrelation mit der Intensität zu erwarten ist, die sich v.a. bei unangenehmen Geruchsbedingungen zeigen sollte.

Vor der fMRT Untersuchung und danach soll eine Befragung hinsichtlich der State- und Trait-Angst mit dem STAI (State-Trait-Angstinventar; Laux, Glanzmann, Schaffner & Spielberger, 1981; Chen, Katdare & Lucas, 2006; A2) erfolgen. Der STAI erfasst über zwei Skalen zu je 20 Items Angst sowohl als Zustand (State), der situationalen Einflüssen unterliegt, als auch Angst als überdauerndes, stabiles Merkmal (Trait). Der STAI soll vor Beginn der Untersuchung (STAI-G Form X-State, Form X2-Trait) eingesetzt werden. Die Bearbeitung der Trait- und State-Skalen des STAI dauert insgesamt etwa 5 Minuten.

Zum Abschluss der fMRT-Messung erfolgt die Aufklärung der Versuchsteilnehmer hinsichtlich Art der Geruchsdarbietung und weitere mögliche Fragen zur Untersuchung werden beantwortet. Die gesamte Untersuchung dauert etwa 45 Minuten.

**3. Verbleib der gewonnen Patienteninformationen**

Die im Rahmen der Analyse gewonnenen Daten werden im Computer und in Stahlschränken des Archivs der Poliklinik für Psychotherapie und Psychosomatik gelagert, die den Datenschutzbestimmungen entsprechen.

**Zielkriterien:**

-Unterschiede in der neuronalen Aktivierung zwischen Gesunden und Patienten mit Panikstörung mit bzw. ohne Agoraphobie bei der Darbietung unterschiedlich valenter Geruchsreize

-Unterschiede in Trait- und State Angst des STAI (Laux, Glanzmann, Schaffner & Spielberger, 1981) zwischen Gesunden und Patienten mit Panikstörung mit bzw. ohne Agoraphobie

-Korrelation zwischen Valenz- und Hedonik-Ratings (A1) und Ausmaß an neuronaler Aktivierung

**Auswertung:**

Grundlage der statistischen Auswertung ist der Vergleich der zwei Untersuchungsgruppen (Patientengruppe, Kontrollgruppe). Die Ergebnisse der Fragebögen-Daten (STAI) liegen im metrischen Datenniveau vor und werden daher zunächst auf das Vorliegen einer Normalverteilung sowie der Gleichheit der Varianzen hin untersucht. Dann erst kann ein Vergleich der Gruppen über t-Tests und Varianzanalyse mit Messwiederholung unter Zuhilfenahme des SPSS 16.0 erfolgen. Die Daten der Nachbefragungen (Ratings) (siehe A1) liegen im ordinalen Datenniveau vor und werden non-parametrisch (U-Test nach Mann und Whitney) ausgewertet.

Die Auswertung der fMRT-Bilder und der klinischen Daten erfolgt mittels des interaktiven Programms SPM 5 (Statistical Parametric Imaging) nach Normalisierung der Daten auf den anatomischen Atlas nach MNI (Talairach und Tournoux, 1988) mittels einer pixel-basierten statistischen Auswertung. Die fMRT-Bilder werden dabei innerhalb der Geruchsbedingungen sowie zwischen den Gruppen (Patienten vs. Gesunde) pro Geruchsbedingung ausgewertet. Darüber hinaus werden Korrelationen der fMRT-Daten mit den Fragebogen-Daten berechnetet.

**Datenschutz:**

Die Fragebogen-Daten (STAI, Nachbefragungen, Dokumentationsbogen) und die fMRT-Daten werden vor der statistischen Auswertung durch eine Codenummer anonymisiert bevor sie in eine passwortgeschützte SPSS-Datei eingegeben werden. Eine Einsichtnahme durch oder die Weitergabe an Dritte ist ausgeschlossen. Zugang zu personengebundenen Daten haben nur die Projektmitarbeiter. Die Patienten erhalten durch die Projektmitarbeiter auf Wunsch eine Rückmeldung zu ihren Untersuchungsergebnissen und beim Vorliegen psychologischer behandlungsbedürftiger Erkrankungen auch Informationen zu Behandlungsangeboten.

**A1: Versuchsprotokoll**

| **Geruch** | **1-8** | **9-16** | **17-24** | **25-32** | **33-40** | **41-48** | **49-56** | **57-64** | **65-72** | **73-80** | **81-88** | **89-96** |  |
| --- | --- | --- | --- | --- | --- | --- | --- | --- | --- | --- | --- | --- | --- |
| **Pfirsich**  **(positiv)** | on | off | on | off | on | off | on | off | on | off | on | off |  |
| **Hydro-gensulfid**  **(negativ)** | on | off | on | off | on | off | on | off | on | off | on | off |  |
| **Körper-schweiß**  **(TSST)** | on | off | on | off | on | off | on | off | on | off | on | off |  |
| **Körper-schweiß**  **(Sport)** | on | off | on | off | on | off | on | off | on | off | on | off |  |

| Rating von Valenz, Intensität und wahrgenommene Angst (mündlich)  STAI-Trait (X2) (Laux et al., 1981)  STAI-G-State (X) (Laux et al., 1981)  on Geruch wird dargeboten  off Geruch wird nicht dargeboten |
| --- |

**Dargestellt ist das on-off-Studiendesign der Geruchspräsentation**

STAI = State Trait Anxiety Inventory

**Reihenfolge der Blöcke:**

1. **P-K-Kst-Ksp**
2. **K-Kst-Ksp-P**
3. **Kst-Ksp-P-K**
4. **Ksp-P-K-Kst**

**P** - Pfirsich

**K** – Künstlicher Schweiß

**Kst** - Körperschweiß (Stress)

**Ksp** - Körperschweiß (Sport)

**A8: Literatur**

Ackerl, K., Atzmueller, M. & Grammer, K. (2002). The scent of fear. Neuroendocrinology Letters, 23, 79-84.

Andy, O. J., Jurko, M. F. & Hughes, J. R. (1975), The amygdala in relation to olfaction. Confinia Neurologica/ Applied Neurophysiology, 37, 215-222.

Bandelow, B., Wedekind, D., Sandvoss, V., Broocks, A., Hajak, G., Pauls, J., Peter, H. & Ruther, E. (2000). Diurnal variation of cortisol in panic disorder. Psychiatry Research, 95 (3), 245-250.

Bartoshuk, L. M. & Beauchamp, G. K. (1994). Chemical Senses. Annual Review of Psychology, 45, 419-449.

Birbaumer, N., Grodd, W., Diedrich, O., Klose, U., Erb, M., Lotze, M., Schneider, F., Weiss, U. & Flor, H. (1998). fMRI reveals amygdala activation to human faces in social phobics. Neuroreport, 9, 1223-1226.

Brand, G. & Millot, J.L. (2001). Sex differences in human olfaction: between evidence and enigma. The Quarterly Journal of Experimental Psychology B. Comparative and Psychology 54, 259–270.

Carr, W. C., Martorano, R. D. & Krames, L. (1970). Responses of mice to odors associated with stress. Journal of Comparative and Physiological Psychology, 71 (2), 223-228.

Chen, D. & Haviland-Jones, J. (2000). Human olfactory communication of emotion. Perceptual & Motor Skills, 91, 771-781.

Chen, D., Katdare, A. & Lucas, N. (2006). Chemosignals of Fear Enhance Cognitive Performance in Humans. Chemical Senses 31(5), 415-423.

Cocke, R. & Thiessen, D. D. (1986). Chemocommunication among prey and predator species. Animal Learning & Behavior, 14, 90-2.

Dickerson, S.S. & Kemeny, M.E., (2004). Acute stressors and cortisol responses: a theoretical integration and synthesis of laboratory research. Psychological Bulletin, 130 (3), 355-391.

Eslinger, P. J., Damasio, A. R. & Van Hoesen, G. W. (1982). Olfactory dysfunction in man: anatomical and behavioral aspects. Brain and Cognition, 1 (3), 259-285.

Etkin, A. & Wager, T. D. (2007). Functional Neuroimaging of Anxiety: A Meta-Analysis of Emotional Processing in PTSD, Social Anxiety Disorder, and Specific Phobia. American Journal of Psychiatry, 164 (10), 1476-1488.

Friston, K. J., Holmes, A. P. & Worsley, K. J. (1999). Comments And Controversies. How Many Subjects Constitute a Study? NeuroImage, 10, 1-5.

Garcia-Leal, C., Parenta, A., Del-Ben, C., Guimarales, F., Moreira, A., Elias, L. & Graeff, F. (2005). Anxiety and salivary cortisol in symptomatic and nonsymptomatic panic patients and healthy volunteers performing simulated public speaking. Psychiatry Research, 133 (2-3), 239-252.

Gottfried, J. A., Deichmann, R., Winston, J. S. & Dolan, R. J. (2002). Functional Heterogeneity in Human Olfactory Cortex: An Event-Related Functional Magnetic Resonance Imaging Study. The Journal of Neuroscience, 22 (24), 10819-10828.

Hinton, D., Ba, P., Peou, S. & Um, K. (2000). Panic Disorder Among Cambodian Refugees Attending a Psychiatric Clinic. Prevalence and Subtype. General Hospital Psychiatry, 22, 437-444.

Hinton, D., Pich, V. & Chhean, D. (2004). Olfactory-Triggered Panic Attacks Among Khmer Refugees: A Contextual Approach. Transcultural Psychiatry, 41 (2), 155-199.

Hinton, D. E., Pich, V., Chhean, D., Pollack, M. H. & Barlow, D. H. (2004). Olfactory-triggered panic attacks among Cambodian refugees attending a psychiatric clinic. General Hospital Psychiatry, 26, 390-397.

Jacob, S. & McClintock, M. K. (2000). Psychological State and Mood Effects of Steroidal Chemosignals in Women and Men. Hormones and Behavior, 37, 57–78.

Kiecolt-Glaser, J. K., Graham, J. E., Malarkey, W. B., Porter, K., Lemeshow, S. & Glaser, R. (2008). Olfactory influences on mood, autonomic endocrine, and immune function. Psychoneuroendocrinology, 33, 328-339.

Kikusui, T. Takigami, S., Tarkeuchi, Y. & Mori, Y. (2001). Alarm pheromone enhances stress-induced hyperthermia in rats. Physiology & Behavior, 72, 45-50.

Kirschbaum, C., Pirke, K. & Hellhammer, D. (1993). The „Trier Social Stress Test“-A tool for investigating psychobiological stress responses in a laboratory setting. Neuropsychobiology, 28, 76-81.

Kohl, J. V., Atzmueller, M., Fink, B. & Grammer, K. (2001). Human Pheromones: Integrating Neuroendocrinology and Ethology. Neuroendocrinology Letters, 22, 309-321.

Leffingwell, J. C. (2002). Olfaction-Update No. 5. Leffingwell Reports, 2 (1), 1-33.

Lorig, T. S. & Roberts, M. (1990). Odor and cognitive alteration of the contingent negative variation. Chemical Senses, 15, 537-545.

Ludewig, S., Geyer, M. A., Ramseier, M., Vollenweider, F. X., Rechsteiner, E. & Cattapan-Ludewig, K. (2005). Information-processing deficits and cognitive dysfunction in panic disorder. Journal of Psychiatry Neuroscience, 30 (1), 37-43

Mackay-Sim, A. & Laing, D. G. (1981). Rats´ responses to blood and body odors of stressed and non-stressed conspecifics. Physiology & Behavior, 27, 503-510.

Martin, G. N. (1996). Olfactory Remediation: Current Evidence And Possible Applications. Social Science & Medicine, 43 (1), 63-70.

Miltner, W., Matjak, M., Braun, C., Diekmann, H. & Brody, S. (1994). Emotional qualities of odors and their influence on the startle reflex in humans. Psychophysiology, 31, 107-110.

Moynihan, J. A., Karp, J. D., Cohen, N. & Ader, R. (2000). Immun deviation following stress odor exposure: role of endogenous opoids. Journal of Neuroimmunology, 102, 145-153.

Pause, B. M., Ohrt, A., Prehn, A. & Ferstl, R. (2004). Positive Emotional Priming of Facial Perception in Females is Diminshed by Chemosensory Anxiety Signals. Chemical Senses, 29, 797-805.

Raudenbush, B., Koon, J., Smith, J. & Zoladz, P. (2003). Effects of odorant administration on objective and subjective measures of sleep quality, post-sleep mood and alertness, and cognitive performance. North American Journal of Psychology, 5, 181-192.

Rolls, E. T., Grabenhorst, F., Margot, C., da Silva, M. A. A. P. & Velazco, M. I. (2008). Selective Attention to Affective Value Alters How the Brain Processes Olfactory Stimuli. Journal of cognitive Neuroscience, 20 (10), pp. 1815–1826.

Rosenzweig, M. R., Breedlove, S. M. & Watson, N. V (2005). Biological Psychology: An Introduction to Behavioral and Cognitive Neuroscience (4th ed.). (Sunderland, MA: Sinaur Associates, Inc).

Sobel, N., Prabhakaran, V., Hartley, C. A., Desmond, J. E., Glover, G. H., Sullivan, E. V. & Gabrieli, D. E. (1999). Blind smell: brain activation induced by an undetected air-borne chemical. Brain, 122, 209-217.

Ströhle, A., Holsboer & F., Rupprecht, R. (2000). Increased ACTH concentrations associated with cholecystokinin tetrapeptide-induced panic attacks in patients with panic disorder. Neuropsychopharmacology, 22 (3), 251-260.

Umweltbundesamt, 2006. Duftstoffe: Wenn Angenehmes zur Last werden kann. Hintergrundpapier. [Online im Internet: URL: <http://www.umweltdaten.de/publikationen/fpdf-l/3550.pdf> (Stand 14.11.2011)].

Talairach, J. & Tournoux, P. (1988). Co-Planar Stereotaxic Atlas of the Human Brain. Stuttgart: Thieme Verlag.

Valenta, J. G. & Rigby, M. K. (1968). Discrimination of the odor of stressed rats. Science, 161, 599-601.

Van den Heuvel, O. A., Veltman, D. J., Groenewegen, H. J., Witter, M. P., Merkelbach, J., Cath, D. C., van Balkom, A. J. L. M., van Oppen, P. & Van Dyck, R. (2005). Disorder-Specific Neuroanatomical Correlates of Attentional Bias in Obsessive-compulsive Disorder, Panic Disorder, and Hypochondriasis. Archives of General Psychiatry, 62, 922–933.

Wedekind, C. & Penn, D. (2000). MHC genes, body odours, and odour preferences. Nephroloy Dialysis Transplantation, 15, 1269-1271.

Wittchen, H. U., Weigel, A. & Pfister, H. (1996). DIA-X — Diagnostisches Expertensystem. Swets Test Services, Frankfurt.

Yousem, D. M., Maldjian, J. A., Siddiqi, F., Hummel, T., Alsop, D. C., Geckle, R. J., Bilker, W. B. & Doty, R. L. (1999). Gender effects on odor-stimulated functional magnetic resonance imaging. Brain Research, 818, 480-487.

Zalaquett, C. & Thiessen, D. (1991). The effects of odors from stressed mice on conspecific behaviour. Physiology & Behavior, 50, 221, 227.

Zald, D. H. & Pardo, J. V. (1997). Emotion, olfaction, and the human amygdala: Amygdala activation during aversive olfactory stimulation. Proceedings of the National Academy of Sciences USA, 94, 4119-4124.

Zald, D. H. & Pardo. J. V. (2000). Functional neuroimaging of the olfactory system in humans. International Journal of Psychophysiology, 36, 165-181.

**A9: Studiendesign**

**fMRT-Messung**

- **Darbietung unterschiedlich valenter Gerüche im on-off Design (A1)**
- **(Pfirsich vs. Künstlicher Schweiß vs. Körperschweißgeruch-TSST vs. Körperschweißgeruch-Sport)**

**Fahrradergometrie**

- **Tragen eines geruchsneutralen T-Shirts**
- **T-Shirt wird bei –80 ° C gelagert**

**Vorgespräch**

**(kurze Aufklärung zur Studie)**

**Diagnostisches Interview**

**(kurze Aufklärung zur Studie)**

**TSST**

**(Trierer Sozial-Stresstest)**

- **Tragen eines geruchsneutralen T-Shirts**
- **T-Shirt wird bei –80 ° C gelagert**

**Interesse an einer Studie zu**

**Stressverarbeitung und Geruchswahrnehmung?**


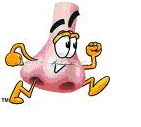


Zur kompletten Untersuchung gehören 4 Termine:

- Absolvierung einer psychologischen Belastungsuntersuchung in Räumen des Uniklinikums an einem Nachmittag (Dauer etwa 2,5 Stunden), währenddessen Blutentnahmen, Messung von Herzfrequenz und Abgabe von Speichelproben zur Bestimmung des Hormons Cortisol
- Überprüfung der Funktionsfähigkeit der körpereigenen Cortisol-Reaktion (Dauer etwa 3 Stunden), währenddessen Blutentnahmen
- Fahrradergometer-Training (Dauer etwa 1 Stunde) mit Messung der Herzrate und Abgabe von Speichelproben zur Bestimmung des Hormons Cortisol
- Termin im Kernspintomografen mit Darbietung von Geruchsproben (Dauer etwa 1 Stunde)

**Voraussetzung:** -Psychisch und körperlich Gesunde

-Patienten mit Angstanfällen

**Aufwandsentschädigung: 150 Euro**

**Bei Interesse melden Sie sich bitte bei:**

Dr. phil. Katja Petrowski/ Dipl.-Psych. Gloria Wintermann

**Telefon:** **0351/** **458 2079**

**fMRT-Riechstudie: Ergometer**

**Datum: Code: Polaruhr:**

**Bitte Pat. bei Ankunft noch mal kurz mit Wasser in den Achselhöhlen waschen lassen!**

|  |  | **Beginn** | **Ende** | **Bemerkungen** |
| --- | --- | --- | --- | --- |
| **Begrüßung**  (5 min) | **Vorgehen erklären**  (10 Minuten Ergometer, vorher und nachher Speichelproben; zwei kurze Fragebögen vor und nach Egometer; T-Shirt anziehen; Brustgurt und Polaruhr umlegen) |  |  |  |
| **Ruhephase** | **Taktatmung erklären**  (5 Sec. einatmen, 5 Sec. ausatmen; 3 Minuten; 6 Atemzüge pro Minute) |  |  |  |
|  | **Marker setzen**  Taktatmung 3 Minuten lang durchführen |  |  |  |
| **1. Speichelprobe**  **-15** | **Marker setzen**  **nach Taktatmung,**  **Sp 15** Minuten **vor** Ergometer  (-15)  **Ca. 3-5 Minuten vor**  **Ergometer:**  **SAM 1**  **STAI –G 1** |  |  |  |
| **2. Speichelprobe**  **-1** | **Marker setzen**  **Sp 1** Minute **vor** Ergometer  (-1) (15 Minuten nach Taktatmung) |  |  |  |
| **Ergometer**  **10 min** | **Marker setzen**  **10 Minuten treten** (Höhe des Ergometers einstellen; Stärke bei 10 Puls bei möglichst mind. 110/max. 120 bpm halten) |  |  |  |
| **3. Speichelprobe**  **+1** | **Marker setzen**  **Sp 1** Minute **nach** Ergometer  (+1)  **SAM 2**  **STAI –G 2** |  |  |  |
| **4. Speichelprobe**  **+10** | **Marker setzen**  **Sp 10** Minuten **nach** Ergometer  (+10) |  |  |  |
| **5. Speichelprobe**  **+20** | **Marker setzen**  **20** Minuten **nach** Ergometer  (+20) |  |  |  |
| **6. Speichelprobe**  **+30** | **Marker setzen**  **30** Minuten **nach** Ergometer  (+30) |  |  |  |
| **Verabschiedung** | Polaruhr/ Brustgurt entfernen  T-Shirt ausziehen |  |  |  |
|  | Verabschiedung |  |  |  |
|  | Kontaktierung für fMRT-Termin erfolgt telefonisch (vorauss. Termin schon mal nennen; Hinweis, dass keine Erkrankungen der Atemwege vorliegen dürfen) |  |  |  |

**Dargestellt ist der Ablauf der Fahrradergometrie-Untersuchung**

SAM = Self Assessment Manikin; STAI = State Trait Anxiety Inventory

**- Hinweis:** Speichelprobe mind. 20 Sekunden im Mund lassen, auf Probe herumkauen, ausreichend einspeicheln
