# Supplementary material for: Cortisol response under low intensity exercise during cognitive-behavioral therapy is associated with therapeutic outcome in panic disorder–an exploratory study
Source: PLoS One. 2022 Sep 1;17(9):e0273413. doi: 10.1371/journal.pone.0273413 (PMC9436097; doi:10.1371/journal.pone.0273413)
Supplement: S3 File — (DOCX) [file pone.0273413.s005.docx]

**S4 File.** List of abbreviations.

AA = Anticipatory Anxiety

ACQ = Agoraphobic Cognitions Questionnaire

ACTH = Adrenocortictropic Hormone

ANCOVA = Analysis of Covariance

AUCg = Area under the response curve with respect to ground

AUCi = Area under the response curve with respect to increase

AV = Agoraphobic Avoidance

BDI = Beck-Depression-Inventory

bpm = beats per minute

BSQ = Body Sensations Questionnaire

CBT = Cognitive-Behavioral Therapy

CRH = Corticotropin-Releasing-Hormone

D = Disability

DSM-IV = Diagnostic and Statistical Manual of Mental Disorders

fMRI = functional magnetic resonance imaging

HC = Health concerns

HPA = Hypothalamic-Pituitary-adrenal

HR = Heart Rate

LIE = Low Intensity Exercise

MI = Mobility Inventory

PA = Panic Attacks

PAS = Panic and Agoraphobia Scale

PD = Panic Disorder

RCT = Randomized Controlled Trials

SAM = Sympathetic-Adreno-Medullar

SAM = Self-Assessment Manikins

SCID = Structured Clinical Interview

STAI = State Trait Anxiety Inventory

TSST = Trier Social Stress Test
