# Supplementary material for: Cortisol response under low intensity exercise during cognitive-behavioral therapy is associated with therapeutic outcome in panic disorder–an exploratory study
Source: PLoS One. 2022 Sep 1;17(9):e0273413. doi: 10.1371/journal.pone.0273413 (PMC9436097; doi:10.1371/journal.pone.0273413)
Supplement: Attachment — Submitted filename: Reply_Wintermann.docx [file pone.0273413.s006.docx]

Dresden, 28.02.2022

Dear editor in chief Jonafer Estoque Miranda,

Thank you for your inquiries and further interest in our present manuscript. We have adhered to your recommendations and revised the manuscript. All changes are marked with yellow.

We have added a report according to the *TREND statement* for improving the reporting quality of nonrandomized evaluations of behavioral interventions (Des Jarlais et al., 2004). Since our trial is not a randomized clinical trial, we cannot apply the CONSORT statement checklist. Nevertheless, we have attached a flow chart (see supplementary material Figure S1).

Furthermore, we have attached a detailed description of the study protocol and recruitment of study participants and included a drop out analysis (see supplementary Table S1). The patients were asked for their participation in a study to investigate the impact of perceiving one´s own stress-related body odors, received during standardized stress conditions, on their neural activations in a fear-network (for a detailed description of the study protocol see supplementary material and Wintermann et al., 2013). We have added this sentence, accordingly, in the manuscript. Above, we have translated the study protocol underlying the ethic votum in English and attached it as supplementary material.

The present trial has not been registered in a clinical register (German Clinical Trials Register) so far because it was only planned as exploratory study. We adhered to your suggestion to register it, despite data aquisition and patient enrolled have been already finished. We also state and confirm that all ongoing and related trials for this intervention are registered in the German Clinical Trials Register.

Please do not hesitate to contact me in case of further inquiries concerning the present manuscript.

Sincerely Yours,

Gloria Wintermann on behalf of all coauthors

References

Des Jarlais, D. C., Lyles, C., Crepaz, N., & the Trend Group (2004). Improving the reporting quality of nonrandomized evaluations of behavioral and public health interventions: The TREND statement. *American Journal of Public Health*, 94,

361-366. For more information, visit: <http://www.cdc.gov/trendstatement/>

Wintermann GB, Kirschbaum C, Petrowski K. Predisposition or side effect of the duration: the reactivity of the HPA-axis under psychosocial stress in panic disorder. International journal of psychophysiology : official journal of the International Organization of Psychophysiology 2016;107:9-15. doi: 10.1016/j.ijpsycho.2016.06.008
