# Supplementary material for: Cortisol response under low intensity exercise during cognitive-behavioral therapy is associated with therapeutic outcome in panic disorder–an exploratory study
Source: PLoS One. 2022 Sep 1;17(9):e0273413. doi: 10.1371/journal.pone.0273413 (PMC9436097; doi:10.1371/journal.pone.0273413)
Supplement: Attachment — Submitted filename: Response to Reviewers.docx [file pone.0273413.s007.docx]

PONE-D-22-01757

Cortisol response under low intensity exercise during cognitive-behavioral therapy is associated with therapy outcome in Panic Disorder – an exploratory study

PLOS ONE

**Dear Dr. Walid Kamal Abdelbasset , Dear editors,**

**thank you for your interest in our manuscript entitled „Cortisol response under low intensity exercise during cognitive-behavioral therapy is associated with therapy outcome in panic disorder – an exploratory study“.**

**We have adhered to your and the reviewers´ inquiries and revised our manuscript accordingly. Please find our point-by-point replies (marked yellow) in the following rebuttel letter. All further changes in the manuscript are coloured in yellow as well.**

**Please do not hesitate to contact me in case of further inquiries.**

**Sincerely Yours,**

**Gloria Wintermann (on behalf of all co-authors)**

Dear Dr. Wintermann,

Thank you for submitting your manuscript to PLOS ONE. After careful consideration, we feel that it has merit but does not fully meet PLOS ONE’s publication criteria as it currently stands. Therefore, we invite you to submit a revised version of the manuscript that addresses the points raised during the review process.

Please submit your revised manuscript by Jun 18 2022 11:59PM. If you will need more time than this to complete your revisions, please reply to this message or contact the journal office at [plosone@plos.org](mailto:plosone@plos.org). When you're ready to submit your revision, log on to https://www.editorialmanager.com/pone/ and select the 'Submissions Needing Revision' folder to locate your manuscript file.

Please include the following items when submitting your revised manuscript:

- A rebuttal letter that responds to each point raised by the academic editor and reviewer(s). You should upload this letter as a separate file labeled 'Response to Reviewers'.

**We have attached the document 'Response to Reviewers' accordingly.**

- A marked-up copy of your manuscript that highlights changes made to the original version. You should upload this as a separate file labeled 'Revised Manuscript with Track Changes'.

**We have attached the document 'Revised Manuscript with Track Changes‘ according to your request.**

- An unmarked version of your revised paper without tracked changes. You should upload this as a separate file labeled 'Manuscript'.

**We have attached the document 'Manuscript‘ according to your request.**

If you would like to make changes to your financial disclosure, please include your updated statement in your cover letter. Guidelines for resubmitting your figure files are available below the reviewer comments at the end of this letter.

If applicable, we recommend that you deposit your laboratory protocols in protocols.io to enhance the reproducibility of your results. Protocols.io assigns your protocol its own identifier (DOI) so that it can be cited independently in the future. For instructions see: <https://journals.plos.org/plosone/s/submission-guidelines#loc-laboratory-protocols>. Additionally, PLOS ONE offers an option for publishing peer-reviewed Lab Protocol articles, which describe protocols hosted on protocols.io. Read more information on sharing protocols at <https://plos.org/protocols?utm_medium=editorial-email&utm_source=authorletters&utm_campaign=protocols>.

**We have decided to allow public access to the study protocol and anonymous research data using the Open Science Framework (OSF)** [**https://osf.io/**](https://osf.io/)**.**

**Access is available using the link:**

**<https://osf.io/s76rw/>**

**and** [**https://archive.org/details/osf-registrations-s76rw-v1**](https://archive.org/details/osf-registrations-s76rw-v1)

**The OSF registration doi is the following: 10.17605/OSF.IO/S76RW**

We look forward to receiving your revised manuscript.

Kind regards,

Walid Kamal Abdelbasset, Ph.D.
Academic Editor
PLOS ONE

Journal Requirements:

When submitting your revision, we need you to address these additional requirements.

1. Please ensure that your manuscript meets PLOS ONE's style requirements, including those for file naming. The PLOS ONE style templates can be found at
   https://journals.plos.org/plosone/s/file?id=wjVg/PLOSOne_formatting_sample_main_body.pdf and 
   <https://journals.plos.org/plosone/s/file?id=ba62/PLOSOne_formatting_sample_title_authors_affiliations.pdf>

**Thank you for this hint. We have adapted the file naming accordingly- both in the text and in the attachments.**

2. Thank you for stating the following financial disclosure:

The present study was funded by the Robert-Pfleger-Stiftung. GBW received the award.
 
Please state what role the funders took in the study.  If the funders had no role, please state: "The funders had no role in study design, data collection and analysis, decision to publish, or preparation of the manuscript." 
If this statement is not correct you must amend it as needed. 
Please include this amended Role of Funder statement in your cover letter; we will change the online submission form on your behalf.

**Please at the financial disclosure as the following statement:**

**Disclosure**

**„The author reports no conflicts of interest in this work. The present study was funded by the Robert-Pfleger-Stiftung. GBW received the award. The funders had no role in study design, data collection and analysis, decision to publish, or preparation of the manuscript.“**

3. Please note that in order to use the direct billing option the corresponding author must be affiliated with the chosen institute. Please either amend your manuscript to change the affiliation or corresponding author, or email us at plosone@plos.org with a request to remove this option.

**The corresponding author Gloria-Beatrice Wintermann is affiliated with the chosen institute.**

4. We note that you have stated that you will provide repository information for your data at acceptance. Should your manuscript be accepted for publication, we will hold it until you provide the relevant accession numbers or DOIs necessary to access your data. If you wish to make changes to your Data Availability statement, please describe these changes in your cover letter and we will update your Data Availability statement to reflect the information you provide.

[Note: HTML markup is below. Please do not edit.]

Reviewers' comments:

Reviewer's Responses to Questions

**Comments to the Author**

1. Is the manuscript technically sound, and do the data support the conclusions?

The manuscript must describe a technically sound piece of scientific research with data that supports the conclusions. Experiments must have been conducted rigorously, with appropriate controls, replication, and sample sizes. The conclusions must be drawn appropriately based on the data presented.

Reviewer #1: Partly

Reviewer #2: Yes

**We have now deposited raw data and supporting information such as the study protocol to a public repository. This will allow to better understand the study design and reproduce the study results.**

2. Has the statistical analysis been performed appropriately and rigorously?

Reviewer #1: Yes

Reviewer #2: I Don't Know

3. Have the authors made all data underlying the findings in their manuscript fully available?

The [PLOS Data policy](http://www.plosone.org/static/policies.action#sharing) requires authors to make all data underlying the findings described in their manuscript fully available without restriction, with rare exception (please refer to the Data Availability Statement in the manuscript PDF file). The data should be provided as part of the manuscript or its supporting information, or deposited to a public repository. For example, in addition to summary statistics, the data points behind means, medians and variance measures should be available. If there are restrictions on publicly sharing data—e.g. participant privacy or use of data from a third party—those must be specified.

Reviewer #1: Yes

Reviewer #2: No

**The study data and further supporting information (the study protocol) are available at a public repository called Open Science Framework (OSF) (see above).**

**The relevant links are the following:**

[**https://osf.io/**](https://osf.io/)**.**

**<https://osf.io/s76rw/>**

[**https://archive.org/details/osf-registrations-s76rw-v1**](https://archive.org/details/osf-registrations-s76rw-v1)

**10.17605/OSF.IO/S76RW**

**All further information needed are available upon request.**

4. Is the manuscript presented in an intelligible fashion and written in standard English?

PLOS ONE does not copyedit accepted manuscripts, so the language in submitted articles must be clear, correct, and unambiguous. Any typographical or grammatical errors should be corrected at revision, so please note any specific errors here.

Reviewer #1: Yes

Reviewer #2: Yes

5. Review Comments to the Author

Please use the space provided to explain your answers to the questions above. You may also include additional comments for the author, including concerns about dual publication, research ethics, or publication ethics. (Please upload your review as an attachment if it exceeds 20,000 characters)

Reviewer #1: The statistical analysis of the study was well thought out and the ANCOVA approach was executed with the limited sample size. Obviously a larger study is needed with up front power calculations and specifically stated objectives. The authors do note that future studies should compare the impact of different intensities of interoceptive exposures, like light vs. moderate or intensive exercise, e.g. using a randomized controlled trial.

**In lines 147-151 we stated the sample size calculation. Study objectives are included on page 4, lines 91-92, page 5, lines 93-94. We also stated that the present study was realized exploratory a spart of another study (see page 5, lines 101-103).**

The investigators gave an honest assessment of the limitations of this study and noted that the present results should be critically reviewed in the context of methodological shortcomings. The sample was not exactly equally paralleled with respect to age and sex. The sample size was quite small with little power, although they do note that a test power of about 34% could be reached when a medium effect size of .25 was supposed (when ANCOVAs were calculated). The present study obviously may be rather evaluated to be a pilot or exploratory study as they note. The descriptive presentation is certainly informative. The investigators should also note with the multiple statistical tests being performed in this limited sample that no adjustment to the p-value has been made for multiple testing and thus the p-values should be interpreted with caution.

**We included a statement that Bonferroni-corrections of p-values were applied where appropriate (see page 11, line 248/ 249). Significance at Bonferroni-adjusted p-value was indicated in the footnote of Table 2 (page 16).**

Reviewer #2: The study titled (Cortisol response under low intensity exercise during cognitive-behavioral therapy is
associated with therapy outcome in Panic Disorder – an exploratory study) is a clinically important one and nicely presented. I have some recommendations:
1- The title: should be "therapeutic" not "therapy"

**We have decided to let the title as it is because the term ‚cognitive-behavioral therapy‘ is well-known by the scientific community in this field. We would be glad if you agreed.**

2- Methods in general lack references in all parts, this is a point of weakness

**Thank you for this hint. We have added lacking references (e.g. for the SCID interview, DSM-IV) and referenced studies the methods applied in the present study were based on (e.g. page 7, lines 141, 143).**

3- Check if unnecessary abbreviations are not needed.

**For economic reasons, we decided to use the abbreviations because. After introducing them, they were well-defined for the readership (e.g. page 7, line 143). We have prepared a list of abbreviations and included it in the supporting information for better understanding the abbreviations (see supporting information S5 File). Furthermore, we omitted any confusing abbreviations, e.g. SAM, which we have formerly used for both Sympathetic-Adreno-Medullar and Self-Assessment Manikins. In the revised version we decided to use the full-term Self-Assessment Manikins (e.g. see Table 1).**

6. PLOS authors have the option to publish the peer review history of their article ([what does this mean?](https://journals.plos.org/plosone/s/editorial-and-peer-review-process#loc-peer-review-history)). If published, this will include your full peer review and any attached files.


If you choose “no”, your identity will remain anonymous but your review may still be made public.


**Do you want your identity to be public for this peer review?** For information about this choice, including consent withdrawal, please see our [Privacy Policy](https://www.plos.org/privacy-policy).

Reviewer #1: No

Reviewer #2: **Yes:** Sawsan Zaitone

[NOTE: If reviewer comments were submitted as an attachment file, they will be attached to this email and accessible via the submission site. Please log into your account, locate the manuscript record, and check for the action link "View Attachments". If this link does not appear, there are no attachment files.]

While revising your submission, please upload your figure files to the Preflight Analysis and Conversion Engine (PACE) digital diagnostic tool, <https://pacev2.apexcovantage.com/>. PACE helps ensure that figures meet PLOS requirements. To use PACE, you must first register as a user. Registration is free. Then, login and navigate to the UPLOAD tab, where you will find detailed instructions on how to use the tool. If you encounter any issues or have any questions when using PACE, please email PLOS at [figures@plos.org](mailto:figures@plos.org). Please note that Supporting Information files do not need this step.

**Figures have been checked according to the tool you suggested (**[**https://pacev2.apexcovantage.com/**](https://pacev2.apexcovantage.com/) **).**
