# Supplementary material for: Cortisol response under low intensity exercise during cognitive-behavioral therapy is associated with therapeutic outcome in panic disorder–an exploratory study
Source: PLoS One. 2022 Sep 1;17(9):e0273413. doi: 10.1371/journal.pone.0273413 (PMC9436097; doi:10.1371/journal.pone.0273413)
Supplement: Attachment — Submitted filename: Rebuttal_Letter_July_2022.docx [file pone.0273413.s008.docx]

PONE-D-22-01757R1

Cortisol response under low intensity exercise during cognitive-behavioral therapy is associated with therapy outcome in panic disorder – an exploratory study

PLOS ONE

Dear Dr. Wintermann,

Thank you for submitting your manuscript to PLOS ONE. After careful consideration, we feel that it has merit but does not fully meet PLOS ONE’s publication criteria as it currently stands. Therefore, we invite you to submit a revised version of the manuscript that addresses the points raised during the review process.

Please submit your revised manuscript by Aug 18 2022 11:59PM. If you will need more time than this to complete your revisions, please reply to this message or contact the journal office at [plosone@plos.org](mailto:plosone@plos.org). When you're ready to submit your revision, log on to https://www.editorialmanager.com/pone/ and select the 'Submissions Needing Revision' folder to locate your manuscript file.

Please include the following items when submitting your revised manuscript:

- A rebuttal letter that responds to each point raised by the academic editor and reviewer(s). You should upload this letter as a separate file labeled 'Response to Reviewers'.
- A marked-up copy of your manuscript that highlights changes made to the original version. You should upload this as a separate file labeled 'Revised Manuscript with Track Changes'.
- An unmarked version of your revised paper without tracked changes. You should upload this as a separate file labeled 'Manuscript'.

If you would like to make changes to your financial disclosure, please include your updated statement in your cover letter. Guidelines for resubmitting your figure files are available below the reviewer comments at the end of this letter.

If applicable, we recommend that you deposit your laboratory protocols in protocols.io to enhance the reproducibility of your results. Protocols.io assigns your protocol its own identifier (DOI) so that it can be cited independently in the future. For instructions see: <https://journals.plos.org/plosone/s/submission-guidelines#loc-laboratory-protocols>. Additionally, PLOS ONE offers an option for publishing peer-reviewed Lab Protocol articles, which describe protocols hosted on protocols.io. Read more information on sharing protocols at <https://plos.org/protocols?utm_medium=editorial-email&utm_source=authorletters&utm_campaign=protocols>.

**We have now published the English study protocol on protocols.io with the following DOI** http://dx.doi.org/10.17504/protocols.io.eq2lynbervx9/v1**. A sentence regarding the publication of the study protocol is included in the revised manuscript, as suggested by PlosOne (see page 6, lines 121-122).**

We look forward to receiving your revised manuscript.

Kind regards,

Walid Kamal Abdelbasset, Ph.D.
Academic Editor
PLOS ONE

**Dear Dr. Walid Kamal Abdelbasset , Dear editors,**

**thank you for your further interest in our manuscript entitled „Cortisol response under low intensity exercise during cognitive-behavioral therapy is associated with therapeutic outcome in panic disorder – an exploratory study“. We have now changed the title according to the reviewer´s suggestion and adapted the term „therapy outcome“ throughout the manuscript. All changes are marked yellow.**

**Please find attached our rebuttal letter with point-by-point replies (also marked yellow).**

**Do not hesitate to contact me in case of further inquiries.**

**Sincerely Yours,**

**Gloria Wintermann (on behalf of all co-authors)**

Journal Requirements:

Please review your reference list to ensure that it is complete and correct. If you have cited papers that have been retracted, please include the rationale for doing so in the manuscript text, or remove these references and replace them with relevant current references. Any changes to the reference list should be mentioned in the rebuttal letter that accompanies your revised manuscript. If you need to cite a retracted article, indicate the article’s retracted status in the References list and also include a citation and full reference for the retraction notice.

[Note: HTML markup is below. Please do not edit.]

Reviewers' comments:

Reviewer's Responses to Questions

**Comments to the Author**

1. If the authors have adequately addressed your comments raised in a previous round of review and you feel that this manuscript is now acceptable for publication, you may indicate that here to bypass the “Comments to the Author” section, enter your conflict of interest statement in the “Confidential to Editor” section, and submit your "Accept" recommendation.

Reviewer #1: All comments have been addressed

Reviewer #2: (No Response)

2. Is the manuscript technically sound, and do the data support the conclusions?

The manuscript must describe a technically sound piece of scientific research with data that supports the conclusions. Experiments must have been conducted rigorously, with appropriate controls, replication, and sample sizes. The conclusions must be drawn appropriately based on the data presented.

Reviewer #1: (No Response)

Reviewer #2: Yes

3. Has the statistical analysis been performed appropriately and rigorously?

Reviewer #1: (No Response)

Reviewer #2: Yes

4. Have the authors made all data underlying the findings in their manuscript fully available?

The [PLOS Data policy](http://www.plosone.org/static/policies.action#sharing) requires authors to make all data underlying the findings described in their manuscript fully available without restriction, with rare exception (please refer to the Data Availability Statement in the manuscript PDF file). The data should be provided as part of the manuscript or its supporting information, or deposited to a public repository. For example, in addition to summary statistics, the data points behind means, medians and variance measures should be available. If there are restrictions on publicly sharing data—e.g. participant privacy or use of data from a third party—those must be specified.

Reviewer #1: (No Response)

Reviewer #2: No

**We have included a statement in the front page of our manuscript and also in the statistical analyses, concerning the availability of our data (see title page, see page 11, lines 258-260).**

**The data file and study protocol are available from the Open Science Framework (**<https://archive.org/details/osf-registrations-s76rw-v1>**, source**  <https://osf.io/s76rw/>**, registration DOI** [**10.17605/OSF.IO/S76RW**](https://doi.org/10.17605/OSF.IO/S76RW) **and identifier DOI 10.17605/OSF.IO/NE4G6).**

5. Is the manuscript presented in an intelligible fashion and written in standard English?

PLOS ONE does not copyedit accepted manuscripts, so the language in submitted articles must be clear, correct, and unambiguous. Any typographical or grammatical errors should be corrected at revision, so please note any specific errors here.

Reviewer #1: (No Response)

Reviewer #2: Yes

6. Review Comments to the Author

Please use the space provided to explain your answers to the questions above. You may also include additional comments for the author, including concerns about dual publication, research ethics, or publication ethics. (Please upload your review as an attachment if it exceeds 20,000 characters)

Reviewer #1: (No Response)

Reviewer #2: The revised version of paper titled (Cortisol response under low intensity exercise during cognitive-behavioral therapy is
associated with therapy outcome in panic disorder – an exploratory study) is improved compared to the original submission.
I find it necessary that authors change (therapy outcome) int he title to (therapeutic outcome)

**Thank you for this hint. We have adhered to your suggestion and replaced the word „therapy“ by „therapeutic“, throughout the whole manuscript, where appropriate.**

7. PLOS authors have the option to publish the peer review history of their article ([what does this mean?](https://journals.plos.org/plosone/s/editorial-and-peer-review-process#loc-peer-review-history)). If published, this will include your full peer review and any attached files.


If you choose “no”, your identity will remain anonymous but your review may still be made public.


**Do you want your identity to be public for this peer review?** For information about this choice, including consent withdrawal, please see our [Privacy Policy](https://www.plos.org/privacy-policy).

Reviewer #1: No

Reviewer #2: No
